# Surface properties and the perception of color

**DOI:** 10.1167/jov.21.2.7

**Published:** 2021-02-12

**Authors:** Zoey J. Isherwood, Quan Huynh-Thu, Matthew Arnison, David Monaghan, Matteo Toscani, Stuart Perry, Vanessa Honson, Juno Kim

**Affiliations:** 1 School of Optometry and Vision Science, University of New South Wales, Sydney, New South Wales, Australia; 2 Department of Psychology, University of Nevada, Reno, NV, USA; 3 Canon Information Systems Research Australia, Macquarie Park, New South Wales, Australia; 4 Nearmap, Sydney, New South Wales, Australia; 5 Canon Information Systems Research Australia, Macquarie Park, New South Wales, Australia; 6 Bandicoot Imaging Sciences, Sydney, New South Wales, Australia; 7 Canon Information Systems Research Australia, Macquarie Park, New South Wales, Australia; 8 Bandicoot Imaging Sciences, Sydney, New South Wales, Australia; 9 Abteilung Allgemeine Psychologie, Justus-Liebig-Universität Giessen, Giessen, Germany; 10 Faculty of Engineering and IT, University of Technology Sydney, Sydney, New South Wales, Australia; 11 School of Optometry and Vision Science, University of New South Wales, Sydney, New South Wales, Australia; 12 School of Optometry and Vision Science, University of New South Wales, Sydney, New South Wales, Australia

**Keywords:** material perception, sensory processes, colour, gloss, 3D shape

## Abstract

We examined whether perception of color saturation and lightness depends on the three-dimensional (3D) shape and surface gloss of surfaces rendered to have different hues. In Experiment 1, we parametrically varied specular roughness of predominantly planar surfaces with different mesoscopic relief heights. The orientation of surfaces was varied relative to the light source and observer. Observers matched perceived lightness and chroma (effectively saturation) using spherical objects rendered using CIE LCH color space. We observed strong interactions between perceived saturation and lightness with changes in surface orientation and surface properties (specular roughness and 3D relief height). Declines in saturation and increases in lightness were observed with increasing specular roughness. Changes in relief height had greater effects on perceived saturation and lightness for blue hues compared with reddish and greenish hues. Experiment 2 found inverse correlations between perceived gloss and specular roughness across conditions. Experiment 3 estimated perceived specular coverage and found that a weighted combination of perceived gloss and specular coverage could account for perceived color saturation and lightness, with different coefficients accounting for the perceptual experience for each of the three hue conditions. These findings suggest that perceived color saturation and lightness depend on the separation of specular highlights from diffuse shading informative of chromatic surface reflectance.

## Introduction

Surfaces reflect light that we use to infer the physical properties of objects (e.g., three-dimensional shape, color, lightness, glossiness). The visual system must recover this information from a finite pattern of luminance variations contained in two-dimensional retinal images. Understanding the underlying processes involved is challenging, because there are instances where the perception of any one surface attribute can be influenced by others (e.g., Marlow, Kim, & Anderson, 2012). In this study, we consider the effects that surface gloss and relief height have on perceptual attributes of surface color.

Evidence from previous literature suggests that color and gloss are co-dependent perceptual constructs that, in combination, affect perceived glossiness, color constancy, and color estimation. For example, perceived gloss can change depending on object color, whereby brighter colors (e.g., yellow) are perceived as less glossy than darker colors (e.g., cyan)—a phenomenon known as contrast gloss (Hunter, 1937; Ferwerda et al. 2001; Baar, Brettel, & Ortiz Segovia, 2015). In other work, increasing the glossiness of an object has been found to improve color constancy, showing that glossy objects have a more stable percept of color under varying illuminations compared to matte objects (Granzier, Vergne, & Gegenfurtner, 2014). Color constancy has also been found to depend on the hue of the object, where blue objects generate greater improvements in color constancy between glossy and matte counterparts compared with red or green objects. Object shape has also been found to play a role whereby smooth shapes have higher color constancy; however, this improvement was only found for the blue and red hues (Granzier et al., 2014). In terms of color estimation, glossiness has also been found to affect the perceived lightness and color saturation of objects (Xiao & Brainard, 2008). Objects appear darker and more saturated with increasing glossiness, with a more pronounced effect for darker colors (e.g., purple) than lighter colors (e.g., yellowish green). Although only a handful of studies have specifically investigated color and shape perception in three-dimensional (3D) objects with both real objects (Giesel & Gegenfurtner, 2010; Granzier et al., 2014; Baar et al., 2015) and rendered objects (Yang & Maloney, 2001; Boyaci, Doerschner, & Maloney, 2004; Fleming, Torralba, & Adelson, 2004; Boyaci, Doerschner, Snyder, & Maloney, 2006; Schultz, Doerschner, & Maloney, 2006; Xiao & Brainard, 2008; Xiao, Hurst, MacIntyre, & Brainard, 2012; Matsumoto, Fukuda, & Uchikawa, 2016), these studies have found that surface color has a profound effect on the perception of other surface properties.

In more recent work on surface properties, Honson, Huynh-Thu, Arnison, Monaghan, Isherwood, and Kim (2020) investigated how object shape, specularity, and surface orientation affect perceived color and gloss. To investigate these material properties, they manipulated the mesoscopic relief height, specular roughness, and orientation of surfaces relative to a primary overhead light source. They measured perceived color saturation and value (of “green” surfaces rendered in hue, saturation, value [HSV] color space) and identified complex interactions among the perception of these color attributes and surface orientation, specular roughness, and surface relief height. Transforming their data to a perceptually uniform CIE LCH color space preserved the effects of specular roughness and relief height on saturation (i.e., C*/L*) and lightness. The authors proposed that the visual misattribution of specular content to diffuse shading could explain the changes in perceived saturation and lightness. Perceived gloss and specular coverage were found to vary in complex ways with changes to specular roughness and mesoscopic relief height. For example, specular coverage was greater when surfaces (and their average surface normals) were oriented at 45° to bisect the angle formed between the viewing and lighting direction. When specular coverage was increased this way, perceived saturation was found to substantially decline. However, when surface relief height was increased, this decline in perceived saturation was limited. This effect of increasing relief on perceived saturation was attributed to reduced specular coverage caused by increased diversity in the range of surface-normal orientations. Despite these complex interactions, the researchers showed that a weighted linear combination of perceived gloss and specular coverage could account for perceived saturation and lightness.

Given the handful of studies conducted in this research area, the observed differences in perceived surface properties (e.g., glossiness, saturation, color) as a function of surface hue are relatively misunderstood. Although differences in perceived surface properties have been observed across a variety of hues, it is particularly notable in many of the aforementioned studies that significant differences were found for bluish hues compared to red and green. There is a large body of evidence indicating that differences in color perception related to the color blue may have to do with perceptual biases along the blue/yellow axis (Churma, 1994; Pearce, Crichton, Mackiewicz, Finlayson, & Hurlbert, 2014; Winkler, Spillmann, Werner, & Webster, 2015; Radonijić et al., 2016; Weiss, Witzel, & Gegenfurtner, 2017; Aston, Radonjić, Brainard, & Hurlbert, 2019). These biases are thought to reflect the presence of colors along the daylight locus in the environment, as natural illumination throughout the day is dominated by blue and yellow light.

Indeed, it has been found that color constancy is worse under blue illumination (Pearce et al., 2014; Winkler et al., 2015), suggesting that the visual system may account for (or rather discount) blue illumination when estimating object hue. This inability to disentangle a blue surface hue from the illuminant in certain situations has been noted across a handful of studies (Churma, 1994; Pearce et al., 2014; Winkler et al., 2015; Radonijić et al., 2016; Weiss et al., 2017; Aston et al., 2019). However, it is relatively unknown to what extent this bias for blue affects perceived surface properties for 3D objects. The majority of research on the blue/yellow perceptual bias has so far only used two-dimensional images to study this phenomenon and/or focused solely on measuring color constancy (Churma, 1994; Pearce et al., 2014; Winkler et al., 2015). Although some studies investigating differences across hues have been conducted using real or rendered 3D objects (Xiao & Brainard, 2008; Giesel & Gegenfurtner, 2010; Xiao et al., 2012; Granzier et al., 2014), it is unclear the extent to which glossiness and specular coverage (as investigated previously in Honson et al., 2020) could account for perceptual differences in saturation and lightness judgments across a wider range of surface and material properties (e.g., mesoscopic relief height, specular roughness, orientation to light source, hue).

To address this gap, here we further evaluated the findings of Honson et al. (2020) by assessing whether the effects of gloss on perceived color saturation and lightness differ across a wider range of hues—red, green, and blue. We measured perceived lightness and saturation (Experiment 1), perceived gloss (Experiment 2), and perceived specular coverage (Experiment 3) for surfaces rendered across these hue conditions at different levels of relief height, specular roughness, and surface orientation relative to the light source. Given that the findings of Honson et al. (2020) may be explained by the inability to disentangle specular content from diffuse shading, we predicted that differences in the pattern of results for perceived lightness and saturation would be observed for blue surfaces but would be similar for red and green surfaces. This difference may be predicted by perceptual biases that exist along the blue/yellow axis that make it difficult for the visual system to disentangle a blue surface hue from the illuminant (Churma, 1994; Pearce et al., 2014; Winkler et al., 2015; Radonijić et al., 2016; Weiss et al., 2017; Aston et al., 2019).

## Experiment 1

The purpose of Experiment 1 was to extend the findings of the original Honson et al. (2020) study to multiple hues. Honson et al. (2020) found that there was large variation in perceived saturation and lightness as a function of changes in specular roughness, mesoscopic shape, and orientation of the planar surfaces to the light source—which can profoundly change the way light interacts with and is distributed across a surface. Specular coverage will be greater when orienting the surface at 45° so its normals bisect the angle formed between the illumination and viewing directions. The increased specular coverage may make perceptual separation of specular and diffuse information more challenging, especially when specular roughness is increased. However, increasing relief height or slanting surfaces more frontally to reduce specular coverage should improve perceptual identification of diffuse color.

Contrary to the view that perceived saturation depends on specular highlight roughness, Xiao and Brainard (2008) found that observer judgments of color appearance were robust against changes in specular parameters between target and matching spheres. In addition, perceived color saturation and lightness were found to be more robust for yellow–green hues compared with purple hues. When viewing objects with more complex structure, such as the tile stimuli used in Honson et al. (2020), specular highlights (among other manipulated factors such as relief height and specular roughness) can greatly distort perceived color appearance. Because Xiao and Brainard (2008) used a less complex stimulus (a smooth sphere), it is possible that this may have played a role in the observed robustness in perceived saturation across specular manipulations. It is also unknown whether the differences that Xiao and Brainard (2008) observed between yellow–green and purple hues would also extend to more complex stimuli.

To ascertain whether biases in perceived lightness are constrained by hue in complex stimuli, we examined the effect of specular roughness and mesoscopic relief height on the perception of saturation and lightness along three distinctly different hue axes. Honson et al. (2020) found that transforming color matching data from the HSV to the CIE LCH color space generated no main effects on perceived chroma (C*), but preserved the effects on perceived lightness and saturation (C*/L*, the ratio of chroma to lightness). In this instance, saturation is the estimated colorfulness of a surface patch that is proportional to the perceived brightness of the patch (Fairchild, 2013). In CIE LCH space, saturation can be computed as C*/L* (Schiller & Gegenfurtner, 2016; Schiller, Valsecchi, & Gegenfurtner, 2018). We used the CIE LCH color space to obtain perceptual estimates of chroma, lightness, and saturation. The CIE LCH color space was used to control the dynamic ranges in saturation and lightness across different hue axes. This allowed us to determine whether there were any differences in the effect of specular roughness and mesoscopic relief height on the appearance of surface colors represented on a standard RGB display.

### Materials and methods

#### Observers

Nine observers with normal or corrected-to-normal vision participated in the experiment, four of whom were authors of the present study (QHT, MA, ZI, DM). All procedures were approved by the Human Research Ethics Advisory Panel at the University of New South Wales.

#### Stimuli

The general process for generating stimuli followed procedures similar to those for the study by Honson et al. (2020). Initially, the upper face of a cube was subdivided into a 203 × 203 vertex mesh. The remaining four vertices that formed the other five faces of the cube were moved toward the upper face to model the profile of a 3D tile 10 cm × 10 cm × 3 cm (width × height × thickness). Mesoscale shape was introduced to the upper face by displacing each vertex along the orthogonal *z*-axis according to a base cloud noise texture map (size, 1.00; Nabla, 0.03; depth, 0) that was generated in Blender 3d 2.77 (Blender Foundation, Amsterdam, The Netherlands). The depth of 0 generated comparatively smoother surfaces than the depth of 1 used by Honson et al. (2020). The values of the displacement map were scaled by different amounts to vary the amplitude of undulations in mesoscopic surface shape along an axis orthogonal to the surface plane. Additional smoothing was performed using the Corrective Smooth modifier in Blender (factor, 1.0; repeat, 12). This smoothing improved the quality of the 3D modeling following displacement mapping. Stimulus images were rendered using an opaque bidirectional scattering distribution function (BSDF) implemented using Cycles render in Blender 3D. The diffuse component was rendered using a Lambertian BSDF, and the specular component was rendered using the microfacet Beckmann function. The number of rays was five for the rendering of each stimulus image.

To investigate how perceived color lightness and chroma are affected by the hue of a surface, surfaces were generated across three different hue angles corresponding to red, green, and blue: 39.999°, 136.016°, and 306.285°, respectively. These hue angles were chosen because they fell on the three primary axes of standard RGB (sRGB) space. The perceptually uniform CIE LCH color space was used to ensure that each hue was perceptually uniform in lightness and chroma—that is, increases in lightness and chroma were perceptually equal across hues. Furthermore, we chose this space because past research has shown that the Lab color space (of which LCH is the polar version) accurately predicts perceived saturation (C*/L*) (Schiller et al., 2018). For luminance around ∼40 cd/m^2^ (middle range of a typical computer monitor), it is the best predicting color space compared to other spaces such as HSV and Luv, and perceived saturation for different hues is on average closer than 1 just noticeable difference to predictions.

The 3D tiles were generated to have a lightness and chroma value fixed at a value of 60 across the three hue angles. Because Blender 3D does not use the LCH color space, LCH values were converted to sRGB values prior to rendering. These values are listed in Table 1 and were converted to sRGB space using custom Python scripts (version 2.7.0) with embedded calls to the colormath library (version 2.2.0; https://github.com/gtaylor/python-colormath).

**Table 1.** LCH and corresponding sRGB values used in Experiment 1.

Hue	LCH	sRGB (0–1)
Red	60°, 60°, 39.999°	0.91, 0.42, 0.31
Green	60°, 60°, 136.016°	0.32, 0.64, 0.26
Blue	60°, 60°, 306.285°	0.63, 0.49, 0.90

**Figure 1.**
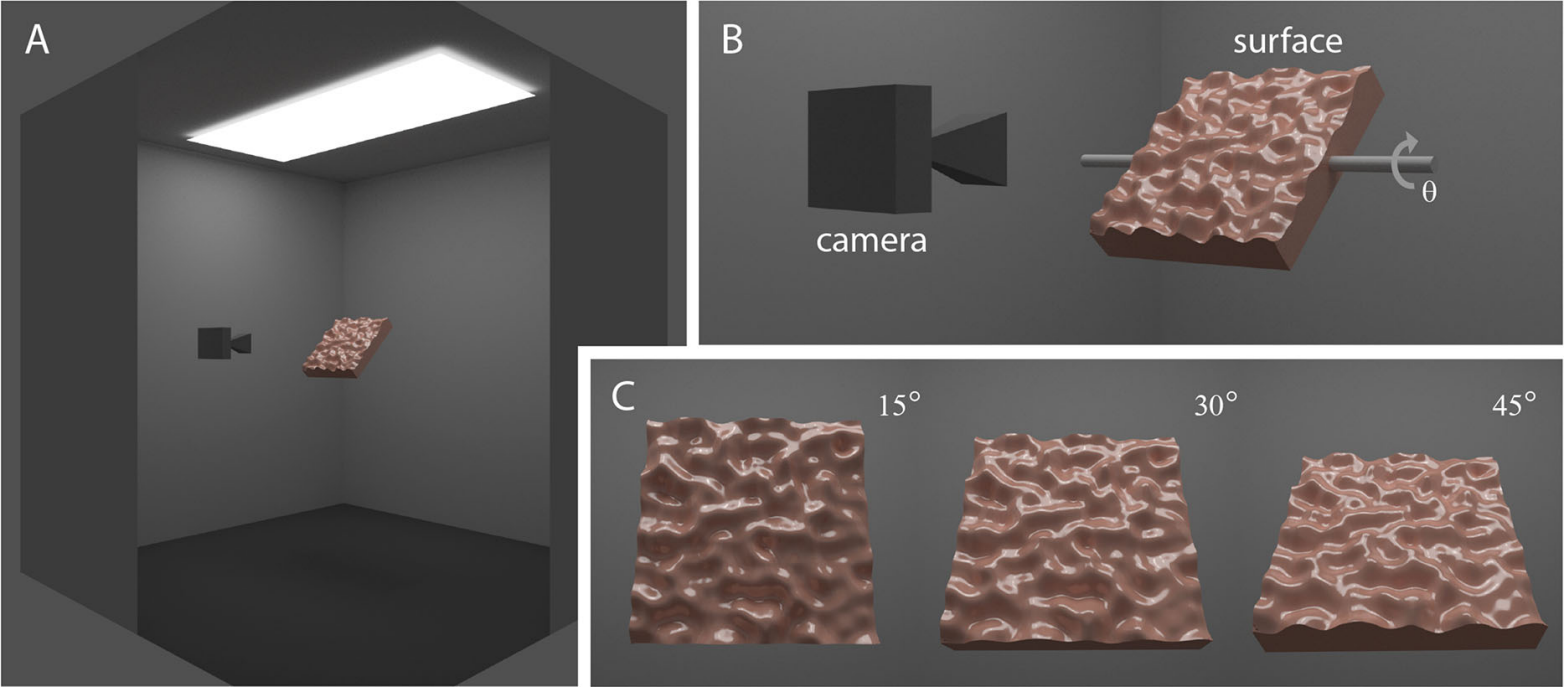
The simulated lighting environment. (A) The lighting environment was an enclosed viewing chamber fitted with an overhead rectangular area light. Note that the near corner of the viewing chamber has been cut away for the purposes of showing the arrangement of overhead lighting, the planar 3D surface (red hue), viewing camera, and internal matte walls (ref = 0.4) and floor (ref = 0.1). (B) Surfaces were slanted obliquely, relative to the viewing direction of the camera, by angular rotations (θ) around the horizontal axis (shown in gray). (C) Sample images showing views of the surface slanted away from frontoparallel to the viewing direction by angles of 15°, 30°, or 45°.

**Figure 2.**
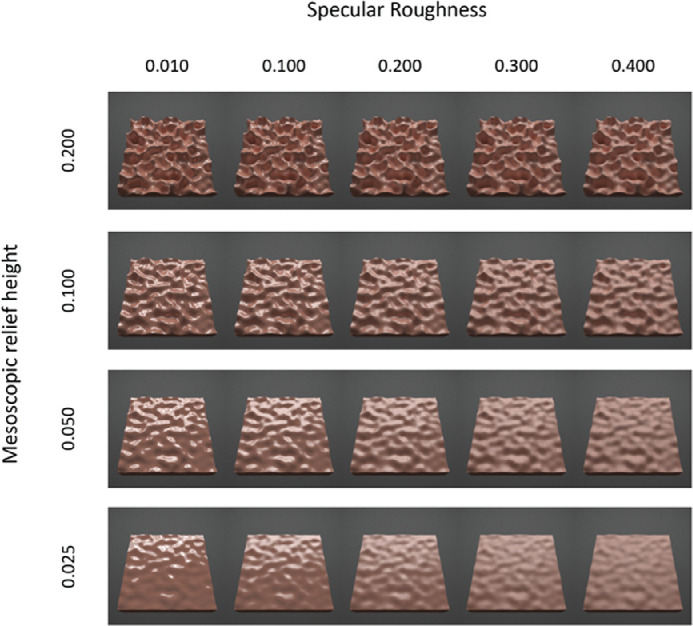
Rendered images of red surfaces varying multiparametrically in mesoscopic shape (across rows) and specular roughness (across columns). Images are shown for the surface oriented at a 30° in slant.

Surfaces were centered within a simulated lighting environment that was similar to standard viewing chambers used in real-world psychophysical experiments on material appearance. Figure 1 provides an overview of the setup for simulated viewing and lighting conditions used in this experiment. The simulated room was a cube (3.28 m^3^) with completely matte walls and floor. We used a large overhead rectangular emitter (2.5 m × 1.0 m) containing an additional two rectangular area lights (6 cm × 120 cm; energy, 100 BLU) to generate natural primary lighting of surfaces embedded in our viewing chamber (Figure 2A). The color of the light was the neutral color of the display (i.e., red = green = blue). A camera with a focal length of 35 mm was situated 60 cm from the midpoint of the surface. This distance was appropriate to ensure that the surfaces remained in full view across changes in their angular orientation of θ around the horizontal *x*-axis (Figure 1B). Figure 1C shows rendered sample images obtained for θ values of 15°, 30°, and 45°.

**Figure 3.**
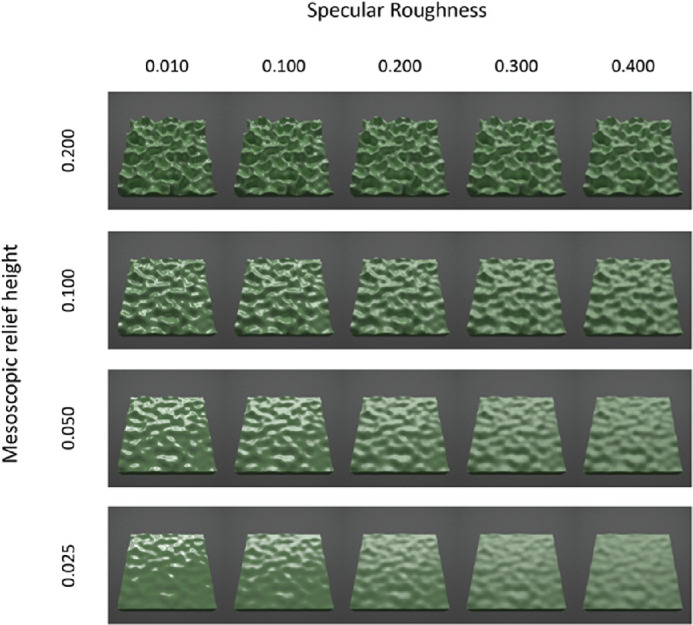
Rendered images of green surfaces varying multiparametrically in mesoscopic shape (across rows) and specular roughness (across columns). Images are shown for the surface oriented at a 30° in slant.

**Figure 4.**
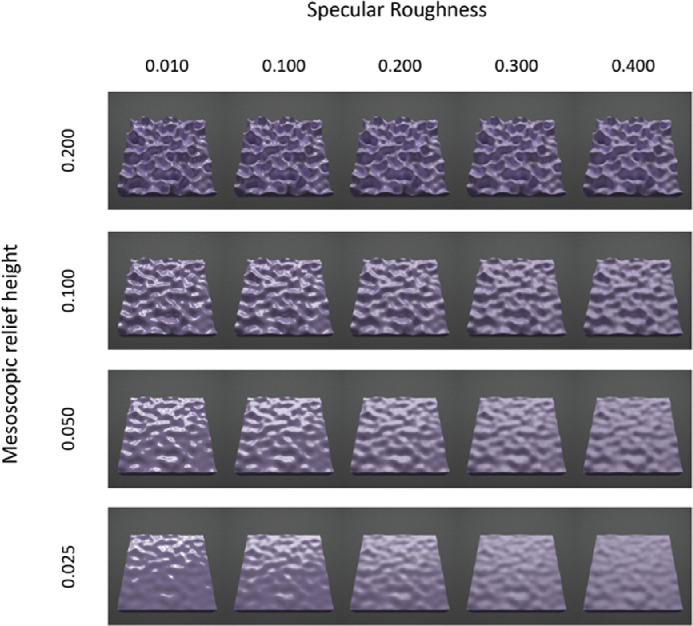
Rendered images of blue surfaces varying multiparametrically in mesoscopic shape (across rows) and specular roughness (across columns). Images are shown for the surface oriented at a 30° in slant.

At each of the three surface orientations, we parametrically varied mesoscopic relief height and specular roughness, as exemplified in the 30° condition for red (Figure 2), green (Figure 3), and blue (Figure 4)surfaces. The complete set of conditions across different surface orientations is provided in Appendix A. We varied mesoscopic relief height over four levels using the vertex displacement modifier in Blender (0.025, 0.050, 0.100, and 0.200). These values scaled the intensity range of the displacement map and generated undulations in mesoscopic shape with peak-to-peak amplitudes that approximately corresponded to 2.5%, 5%, 10%, and 20% of the width of the surface. We also parametrically varied specular roughness over five levels (0.010, 0.100, 0.200, 0.300, and 0.400) while holding specular amplitude at 0.2 in the simulation. This range of specular roughness levels was chosen to be similar to ranges used in previous research (Mooney & Anderson, 2014; Honson et al., 2020). Specular amplitude was held constant at 0.20, as used previously to generate the realistic glossy appearance of common natural materials (e.g., Marlow et al., 2012).

Images were rendered for each stimulus conditions at a resolution of 2000 × 2000 pixels in 24-bit RGB bitmap format. The rendered images were generated using the Cycles Render for Blender 2.77, which was controlled by a custom Python script running on a Dell Precision 5510 (Dell Technologies, Round Rock, TX) with an Intel Core i7-6820HQ CPU at 2.70 GHz (Intel Corporation, Santa Clara, CA). Path tracing was used with 128 render samples per pixel. The simulated light paths were set with default parameters for full global illumination. These rendering parameters were appropriate for generating images that could be subsampled to 800 × 800 using the Lanczos filter method to eliminate noise. Custom stimulus presentation software was used to present these images on an Eizo ColorEdge CG275W monitor (27-inch diagonal with resolution 1920 × 1200, sRGB color mode, 8 bits per channel, 2.2 gamma, D65 color temperature; Eizo Nanao Corporation, Ishikawa, Japan). Images were viewed at a distance of approximately 70 cm for an effective size of approximately ±10° visual angle (horizontal and vertical). The estimated luminance from sRGB values computed over a square region of the surface images for the three different hues was roughly equal: 40.6 (red), 41.9 (green), and 42.4 (blue).

#### Procedure

Prior to participating, observers were informed that they would be required to make perceptual matches of surface color for planar surface images that were presented in a random order on a computer monitor. Training was offered for some observers to gain familiarity with what they were required to match. The pre-rendered images used in training were of a smooth planar surface devoid of mesoscopic surface changes presented on the left side of the display. Most of the observers were confident that they understood the task after completing several trials. For the actual experiment, three blocks (each with a different hue condition) of 60 images were presented in a randomized order on the left side of the display (four levels of relief height, five levels of specular roughness, and three levels of orientation relative to the light source). The order of blocks was pseudorandomized across observers for performing the matches to each of the three hue conditions. A short rest break of up to 5 minutes was provided between blocks. The entire experiment took each of the participants no longer than 1 hour to complete.

**Figure 5.**
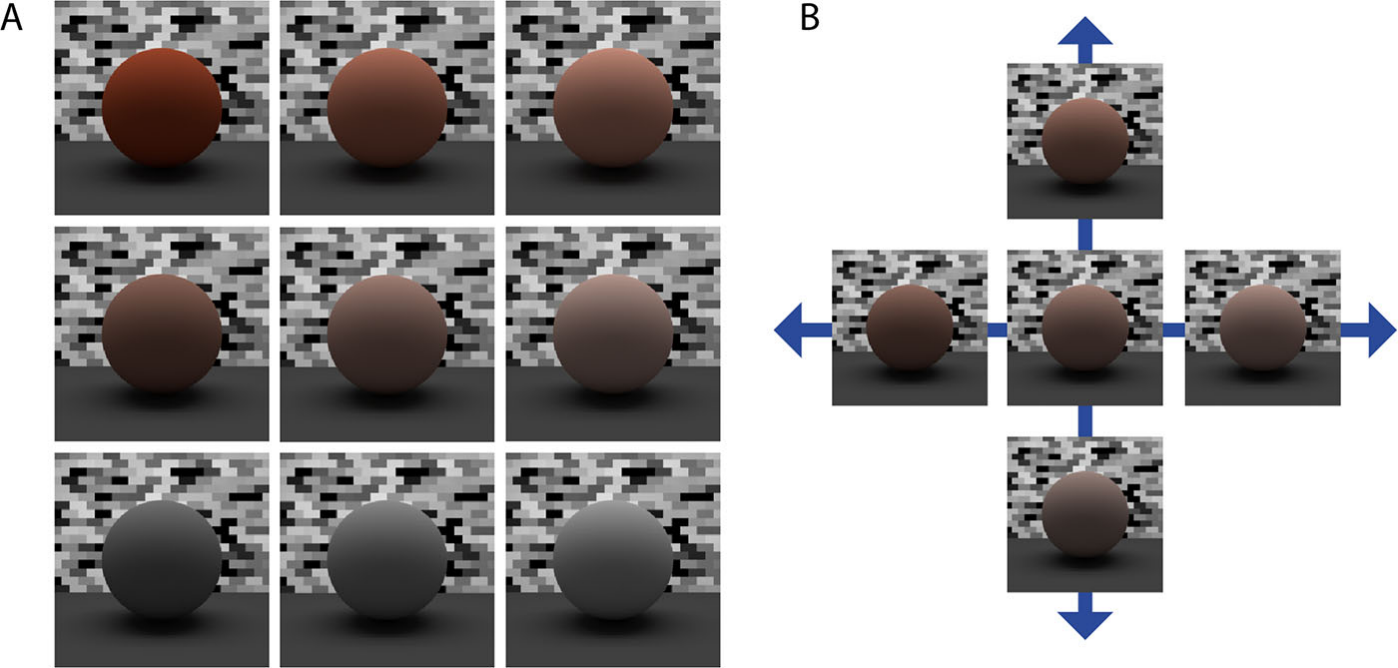
Spherical surfaces were used in the asymmetric color matching task. (A) The surfaces were situated within the same light field as the bumpy planar surfaces, but with a mural of random brick texturing situated on the far wall behind the spheres. The sphere was also rendered on a flat plane that provided some ambient lighting to the underside of the sphere to increase the realism of the display and accurate perception of shape. (B) Selections were varied by preset steps in chroma (along columns) and lightness (along rows).

**Figure 6.**
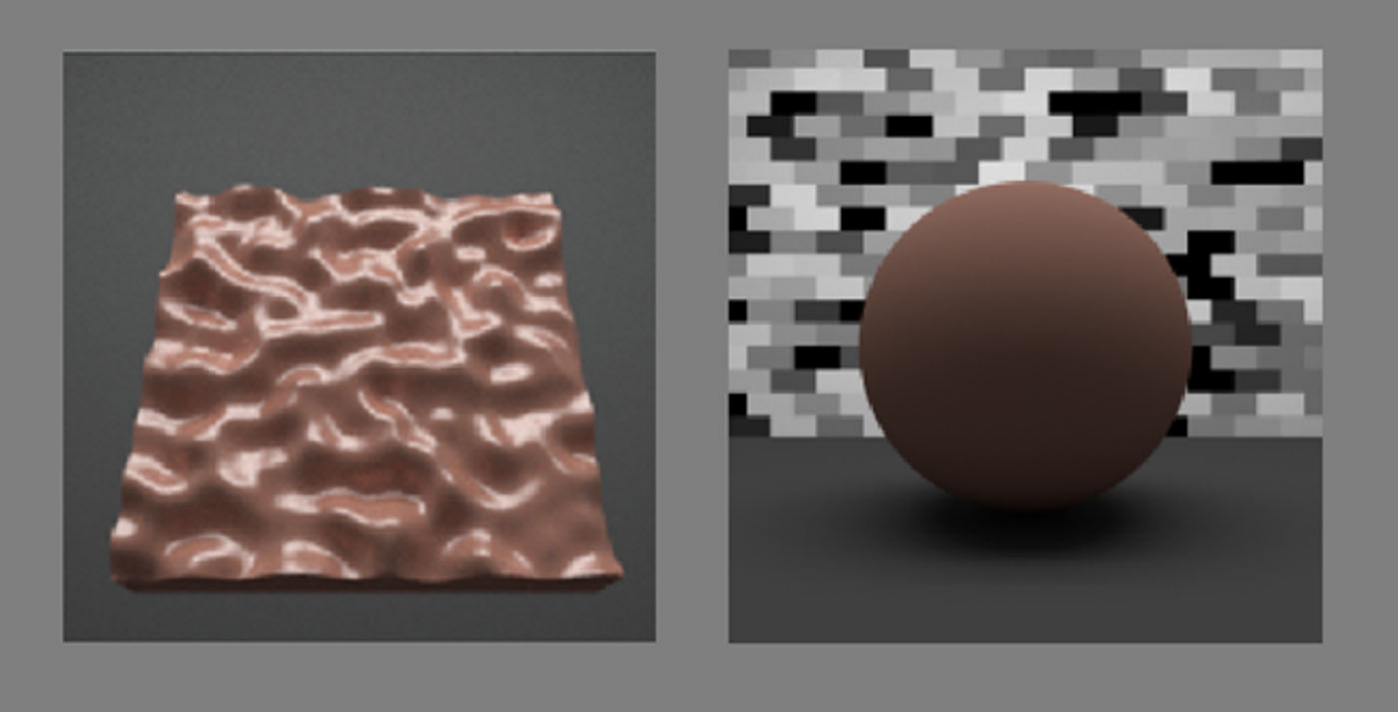
Stimulus presentation and feedback. In this example of a single experimental trial, a randomly presented plane is situated on the left of the display. The observer's task was to adjust the lightness and chroma of the matte sphere on the right to match the plane.

Perceptual matches of lightness and chroma were made using pre-rendered images of a matte sphere devoid of specular reflections that was presented on the right side of the display (Figure 5). We used a sphere to ensure that the distribution of surface orientations was compatible with all three surface orientations of target planes. A sample scenario of the matching task is shown in Figure 6. Observers used the arrow keys on a standard keyboard to move through a pre-rendered 22 × 22 matrix of images (22 levels of lightness and 22 levels of chroma). The hue of matching spheres was the same as the target surface. The LCH space was used to control the changes in lightness and chroma of the matching sphere to ensure that each step would be perceptually uniform across hues. Horizontal keypresses increased or decreased lightness (ranging from 18.750 to 97.500; step size, 3.75). Vertical keypresses increased or decreased chroma (ranging from 22.500 to 101.250; step size, 3.75). The observer pressed the spacebar to record the setting that appeared to most closely match the lightness and chroma of the target plane. Responses were recorded to ASCII files for subsequent data analysis. We used a higher lightness (97.5) and chroma (101.25) than the values used to generate the tiles (60.0) because Honson et al. (2020) found saturation to be perceived up to ∼10% higher than the simulated saturation when matching with matte spheres, depending on orientation, relief height, and specular roughness. We verified that the monitor could display these extended LCH combinations by converting the values to an sRGB color space to and ensuring these values were within sRGB bounds.

#### Data analysis

Observer settings of chroma and lightness were separately averaged across observers for plotting purposes. A four-way, repeated-measures analysis of variance (ANOVA) was used to test for main effects of specular roughness, relief height, and surface orientation on perceived chroma and lightness. The analysis was performed separately on the three different hue conditions.

### Results and discussion

**Figure 7.**
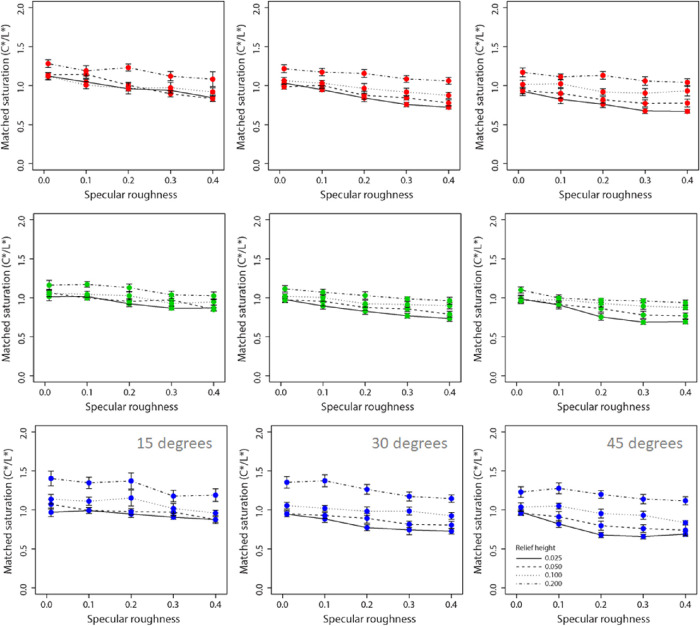
Mean ratings of perceived saturation (C*/L*) for red (upper row), green (middle row), and blue (lower row) surfaces plotted as a function of specular roughness. Different lines are used to represent each relief height and separate axes, and data are shown for the 15°, 30°, and 45° surface orientations.

**Figure 8.**
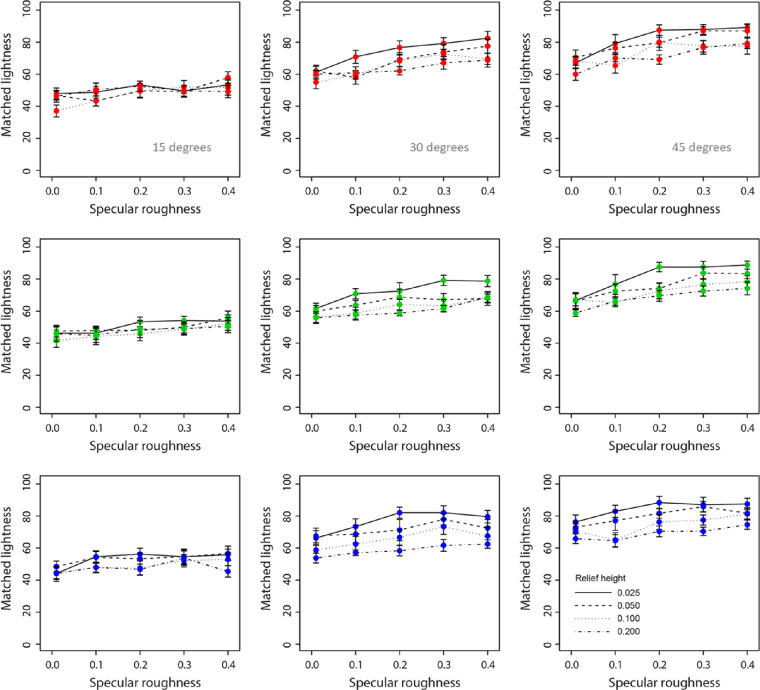
Mean ratings of perceived lightness for red (upper row), green (middle row), and blue (lower row) surfaces plotted as a function of specular roughness. Different lines are used to represent each relief height and separate axes, and data are shown for the 15°, 30°, and 45° surface orientations.

Figures 7 and 8 show data for perceived chroma and lightness, respectively. We observed a very slight decline in chroma with increased specular roughness across the three hue conditions. There appears to be a clear increase in lightness with increasing specular roughness across all hue conditions.

#### Perceived saturation (C*/L*)

A four-way, repeated-measures ANOVA found a significant main effect of hue on perceived saturation, *F*(2, 16) = 4.09, *p* < 0.05. There were also main effects of surface orientation, *F*(2, 16) = 82.99, *p* < 0.00001, and relief height, *F*(3, 24) = 71.10, *p* < 0.00001, on perceived saturation. There was also a significant main effect of specular roughness of perceived saturation, *F*(4, 32) = 87.12, *p* < 0.00001. There was a significant two-way interaction effect between hue and relief height on perceived saturation, *F*(6, 48) = 21.89, *p* < 0.00001. There was a significant two-way interaction effect between surface orientation and relief height on perceived saturation, *F*(6, 48) = 4.62, *p* < 0.001. There was also a significant two-way interaction effect between relief height and specular roughness on perceived saturation, *F*(12, 96) = 3.09, *p* < 0.001. We also found a significant three-way interaction effect across hues in the relationship between relief height and specular roughness on perceived saturation, *F*(12, 96) = 2.16, *p* < 0.01. There were no other interaction effects on perceived saturation.

The results suggest that viewing orientation plays an important role in the perception of color saturation, with obliquely oriented surfaces generating lower levels of perceived saturation than more frontally oriented surfaces. There was a clear and consistent effect of relief height on perceived saturation across all conditions, with greater relief generating higher perceived saturation. Overall, there was a consistent inverse association between specular roughness and perceived saturation, as increasing specular roughness was found to reduce perceived saturation. This effect of specular roughness on perceived saturation was found to be consistent across the three hues, but the effect of relief height was differential; the effects of relief height on perceived saturation were greatest for the blue hue and lowest for the green hue. This was confirmed by the signification two-way interaction effect between hue and relief height on perceived saturation. Further, the three-way interaction effect suggests that the effect of specular roughness of perceived saturation varies nonlinearly with relief height, and the extent of this nonlinearity differs across hues.

#### Perceived lightness

A four-way, repeated-measures ANOVA found a significant main effect of hue on perceived lightness, *F*(2, 16) = 5.58, *p* < 0.05. There were also main effects of surface orientation, *F*(2, 16) = 91.65, *p* < 0.00001, and relief height on perceived lightness, *F*(3, 24) = 21.30, *p* < 0.00001. There was also a significant main effect of specular roughness on perceived lightness, *F*(4, 32) = 47.65, *p* < 0.00001. There was a significant two-way interaction effect between hue and relief height on perceived lightness, *F*(6, 48) = 3.58, *p* < 0.01. There was a significant two-way interaction effect between surface orientation and relief height on perceived lightness, *F*(6, 48) = 5.76, *p* < 0.0005. There was a significant two-way interaction effect between surface orientation and specular roughness on perceived lightness, *F*(8, 64) = 3.90, *p* < 0.001. There was also a significant two-way interaction effect between relief height and specular roughness on perceived lightness, *F*(12, 96) = 2.47, *p* < 0.01. We also found a significant three-way interaction effect across hues in the relationship between surface orientation and specular roughness on perceived lightness, *F*(16, 128) = 1.81, *p* < 0.05. There was also a significant three-way interaction effect across surface orientations in the relationship between surface relief height and specular roughness on perceived lightness, *F*(24, 192) = 1.72, *p* < 0.05. No other interaction effects on perceived lightness were found.

For lightness matches, across the three hues we observe a consistent linear increase in matched lightness with increasing specular roughness, following the findings of Honson et al. (2020). This increase in matched lightness is consistent across all relief heights and orientation conditions, the only difference being the finding that the magnitude of the effect monotonically increased with higher roughness and orientation values (e.g., 0.4 roughness at 45°). The magnitude of this monotonic increase in matched lightness significantly differed across hues. This difference was observed in the significant two-way interactions between relief height and specular roughness, as well as between relief height and surface orientation. The significant three-way interactions suggest that the effect of specular roughness on perceived lightness varies as a function of relief height, and this relationship varies with either surface orientation or hue. Perceived lightness was more consistent across changes in relief height and specular roughness for red surfaces and lowest in consistency across these conditions for blue surfaces. These effects depended on viewing orientation, with more frontally aligned surfaces generating lower variation in perceived lightness with relief height across levels of specular roughness for all hues.

Taken together, these data suggest that perceived saturation and lightness were most distorted when relief height was low and specular roughness was high, whereby the stimulus is perceived to have lower saturation and higher lightness than the true values (lightness, 60; chroma, 60). Interestingly, the magnitude of this effect was largest for blue surfaces and was relatively similar between red and green. Thus, the question arises: Why do large differences occur only for blue?

One may argue that the differences we observed may be due to sRGB gamut limits; that is, observers would choose settings that are out of gamut and thus artificially create hue differences, as the limits for L* and C* depend on H*. To figure out whether this was the case, we have plotted in the left panel of Appendix B the C* limit (max C*) for each L* level for the three H*values we used (i.e., the largest C* value at which the corresponding RGB triplet has values between 0 and 1). The red hue has the smallest range (Appendix B, left panel) to select from, which would predict large ceiling effects for red when estimating lightness; however, this was not the case in our results (see Figure 8). On the right panel of Appendix B, we have plotted the saturation limit (max S) as a function of L* for the three hues. The borders differ, potentially creating gamut artifacts. We have also plotted the maximum saturation settings (from the data plotted in Figure 7) with corresponding L* levels in the right panel of Appendix B (from the data plotted in Figure 8; green circle for the green hue; red, ×; blue, +). The three maxima nearly overlap, indicating that differences between hues are not caused by different upper limits of saturation. To compute the limit, for each L* level (0 to 100) and each of the three H* values used in the present study, we input a broad range of C* values converted to sRGB and looked at the maximum C* values produced within the sRGB gamut. Although the green hue gets close to the maxima, because the highest saturation settings overlap and do not touch the upper limits, this suggests that our results were not driven by differences in the upper limits of different hues.

So, what else can account for the differences in lightness and saturation judgments observed for blue? It is possible that the different pattern of results we observed for blue may be due to perceptual biases along the blue/yellow axis (Churma, 1994; Pearce et al., 2014, Winkler et al., 2015; Radonijić et al., 2016; Weiss et al., 2017; Aston et al., 2019). Past research has found that that color constancy is worse when a scene is illuminated by blue light (Pearce et al., 2014; Winkler et al., 2015), and that it is difficult for the visual system to disentangle blue surface hues from the illuminant in certain scenarios (Churma, 1994; Pearce et al., 2014; Winkler et al., 2015). For example, Winkler et al. (2015) found that people tend to attribute bluish tints to the illuminant rather than the object itself.

The findings of these past studies suggest that the visual system has a prior for discounting/accounting for blue illumination when estimating surface hue. In the present study, given the large differences in the way the light source illuminates the tile stimuli across orientations, mesoscopic relief heights, and specular roughness levels, it is likely that the process of disentangling the illuminant from the tiles when judging lightness and saturation would substantially differ for blue hues compared to red and green. When estimating lightness, this inability to disentangle the illuminant from the tiles themselves may be the reason why lightness judgments were inflated for blue tiles with low relief heights. Specifically, it has been demonstrated by Toscani et al. (2013) that the lightness of an object is determined by what is deemed as the brightest part of the object—which can be a problem if the illuminant cannot be correctly parsed from the object hue.

Although perceived saturation was variable across the three hues, the magnitude of the effect of decreasing saturation with increasing specular roughness is extremely subtle. These data are consistent with the dominant response in lightness and saturation based on color matching data transformed from HSV to CIE LCH space by the Honson et al. (2020) study. Across all surface images, we found a systematic effect of relief height on perceived saturation and lightness. This effect would appear to suggest that increases in the distribution of specular reflections and not only their clarity (i.e., roughness) are important for estimating surface saturation and lightness. The effect of surface relief height on perceived color attributes is consistent with our previous research (Honson et al., 2020). We therefore examined the effect of relief height and specular roughness on perceived gloss and specular coverage in the experiments that follow.

## Experiment 2

The previous experiments found that chroma and lightness judgments were strongly dependent on specular roughness, mesoscopic relief, and surface orientation. Although perceived lightness was found to increase significantly with increasing specular roughness, perceived saturation declined more for the short-wavelength surface (blue hue) and less for red and even less for green. The results of Experiment 1 followed the findings of Honson et al. (2020), whereby perceived lightness was farthest from veridical when specular roughness was high and mesoscopic relief height was low. It may be possible that these differences arise due to the incomplete separation of specular reflections from diffuse shading when specular edges have increased roughness. If some of the specular energy were (mis-)classified as diffuse shading in this way, then proportionally less classifiable specular content would be available for generating the experience of gloss. Although differences in perceived lightness were subtle across hue, it may be possible that differences in this incomplete separation may explain these effects on perceived color. In Experiment 2, we investigated whether hue affects perceived gloss across changes in specular roughness, mesoscopic relief, and surface orientation.

### Materials and methods

#### Observers

Four observers with normal or corrected-to-normal vision were recruited. All participants were authors of this study and were well trained in performing psychophysical tasks. All procedures received local ethical clearance and followed the principles outlined in the Declaration of Helsinki.

#### Stimuli

We used planar surface images that were identical to those used in Experiment 1. However, because we used the paired-comparisons method here, we eliminated the 0.200 specular roughness value to reduce the number of trials. We eliminated this particular specular roughness value based on the findings of Honson et al. (2020), because nonlinearities in perceived gloss were not present between specular roughness values 0.010 and 0.300. As such, the conditions for specular roughness were 0.010, 0.100, 0.300, and 0.400; for relief height, they were 0.025, 0.050, 0.100, and 0.200. The same orientation (15°, 30°, and 45°) and hue (red, green, blue) conditions were used as in Experiment 1.

#### Procedure

Following Honson et al. (2020), we measured perceived surface gloss using the paired-comparisons method. Observers were asked to select which of two images presented side-by-side appeared glossier or shinier using the left/right arrow keys on the keyboard. The terms glossier or shiner were used to ensure that observers were attending to the overall specular component and not merely the material quality of the surface. Observer responses were recorded to an ASCII file for subsequent analysis. To minimize the number of trials in a session, we broke up the experiment into nine blocks: one for each level of surface orientation (15°, 30°, and 45°) and hue (red, green, and blue). Within each block there were 240 trials based on the four levels of specular roughness and four levels of relief height (16 × 16–16 conditions). We focused our comparisons on relief height and specular roughness, as these conditions were found to generate the strongest main effects in Experiment 1. Image pairs were fully randomized, and we pseudorandomized the order for performing blocks of trials at each surface orientation and hue across observers. Although the paired images were presented for an unlimited period of time, observers tended to make their judgments within approximately 5 seconds. Observers took between 1.5 and 2.0 hours to complete the experiment, and all nine blocks were either completed in one session with short breaks or over a period of 2 days.

#### Data analysis

As in Honson et al. (2020), we computed probability estimates of perceived gloss for each image in each condition by dividing the number of times the image was selected as glossier by the number of times it was presented on the display. Probability estimates of perceived gloss were analyzed using a series of repeated-measures, two-way ANOVAs in R (R Foundation for Statistical Computing, Vienna, Austria).

### Results and discussion

**Figure 9.**
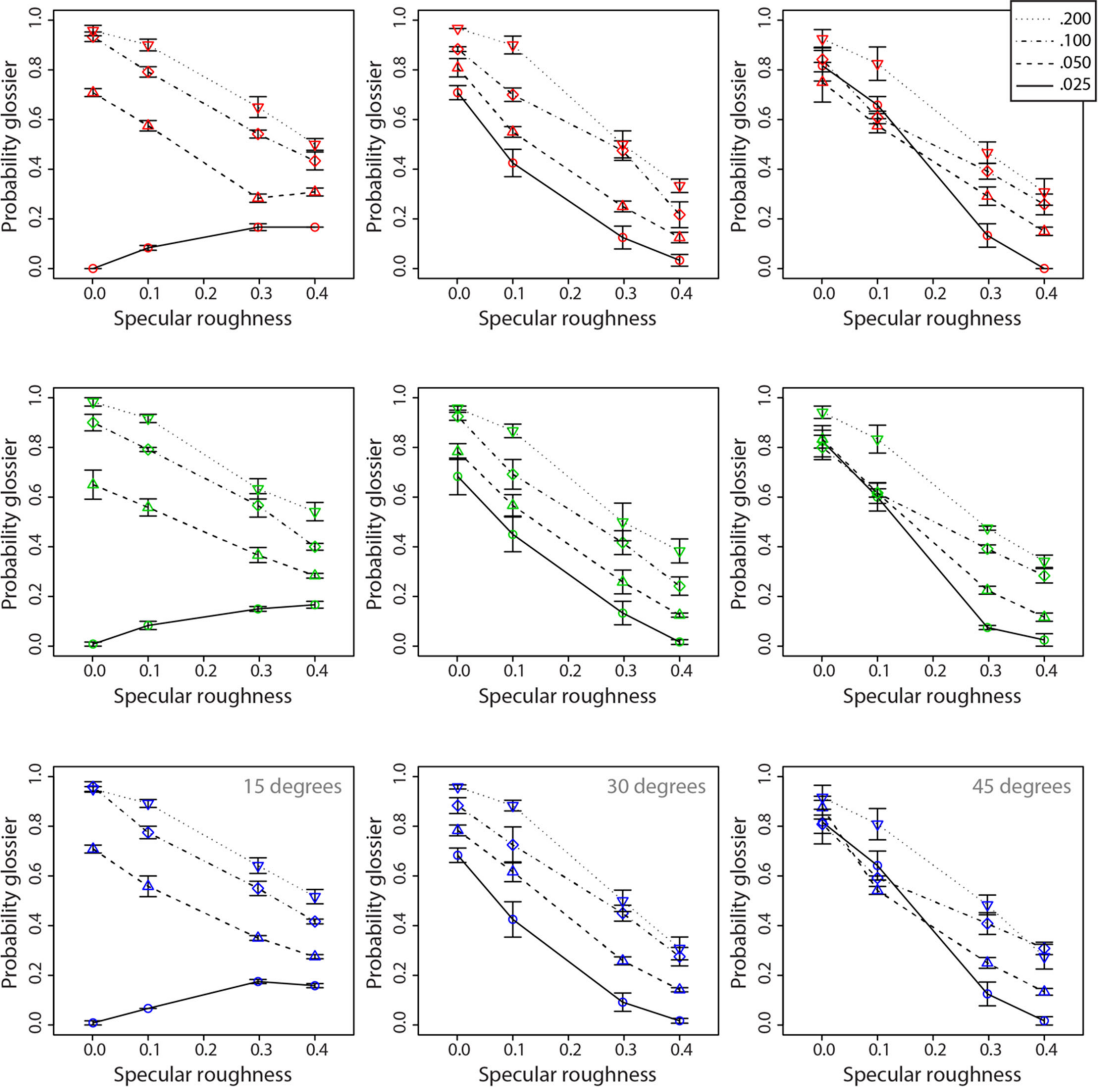
Means and standard errors of probability estimates for perceived gloss of planar surfaces varying in specular roughness and relief height across three different hues: red, green, and blue (top, middle, and bottom rows, respectively). Data plotted in each column represent the three surface orientations used in the experiment (15°, 30°, and 45°). Different line types and symbols are shown in the legend and correspond to data across the different relief heights.

Perceived gloss was plotted as a function of specular roughness for each of the levels of relief height and viewing orientation (Figure 9). Separate rows of axes show data obtained for surfaces having each of the three different diffuse colors. We analyzed the results separately for each of the nine conditions below.

Red surfaces were analyzed for effects on perceived gloss at each viewing orientation using separate repeated-measures ANOVAs. For the 15° condition, there was a significant main effect of specular roughness, *F*(3, 9) = 244.2, *p* < 0.00001, and relief height, *F*(3, 9) = 281.7, *p* < 0.0005. There was also a significant interaction effect between specular roughness and relief height on perceived gloss, *F*(9, 27) = 77.44, *p* < 0.0005. For the 30° condition, there was a significant main effect of specular roughness, *F*(3, 9) = 320.6, *p* < 0.00001, and relief height, *F*(3, 9) = 24.52, *p* < 0.0005. There was also a significant interaction effect between specular roughness and relief height on perceived gloss, *F*(9, 27) = 5.65, *p* < 0.0005. For the 45° condition, there was a significant main effect of specular roughness, *F*(3, 9) = 180.7, *p* < 0.00001, and relief height, *F*(3, 9) = 9.01, *p* < 0.005. There was also a significant interaction effect between specular roughness and relief height on perceived gloss, *F*(9, 27) = 5.65, *p* < 0.0005.

Next, green surfaces were analyzed for effects on perceived gloss at each viewing orientation using separate repeated-measures ANOVAs. For the 15° condition, there was a significant main effect of specular roughness, *F*(3, 9) = 114.8, *p* < 0.00001, and relief height *F*(3, 9) = 165.1, *p* < 0.00001. There was also a significant interaction effect between specular roughness and relief height on perceived gloss, *F*(9, 27) = 32.97, *p* < 0.00001. For the 30° condition, there was a significant main effect of specular roughness, *F*(3, 9) = 159.9, *p* < 0.00001, and relief height, *F*(3, 9) = 13.88, *p* < 0.005. However, there was no significant interaction effect between specular roughness and relief height on perceived gloss, *F*(9, 27) = 1.22, *p* < 0.32. For the 45° condition, there was a significant main effect of specular roughness, *F*(3, 9) = 693, *p* < 0.00001, and relief height, *F*(3, 9) = 24.29, *p* < 0.0005. There was also a significant interaction effect between specular roughness and relief height on perceived gloss, *F*(9, 27) = 4.47, *p* < 0.005.

Finally, blue surfaces were analyzed for effects on perceived gloss at each viewing orientation using separate repeated-measures ANOVAs. For the 15° condition, there was a significant main effect of specular roughness, *F*(3, 9) = 438, *p* < 0.00001, and relief height, *F*(3, 9) = 165.4, *p* < 0.00001. There was also a significant interaction effect between specular roughness and relief height on perceived gloss, *F*(9, 27) = 70.25, *p* < 0.00001. For the 30° condition, there was a significant main effect of specular roughness, *F*(3, 9) = 169.2, *p* < 0.00001, and relief height, *F*(3, 9) = 33.22, *p* < 0.00005. However, there was no significant interaction effect between specular roughness and relief height on perceived gloss, *F*(9, 27) = 2.07, *p* < 0.07. For the 45° condition, there was a significant main effect of specular roughness, *F*(3, 9) = 404.1, *p* < 0.00001, and relief height, *F*(3, 9) = 5.89, *p* < 0.05. There was also a significant interaction effect between specular roughness and relief height on perceived gloss, *F*(9, 27) = 7.37, *p* < 0.00005.

**Figure 10.**
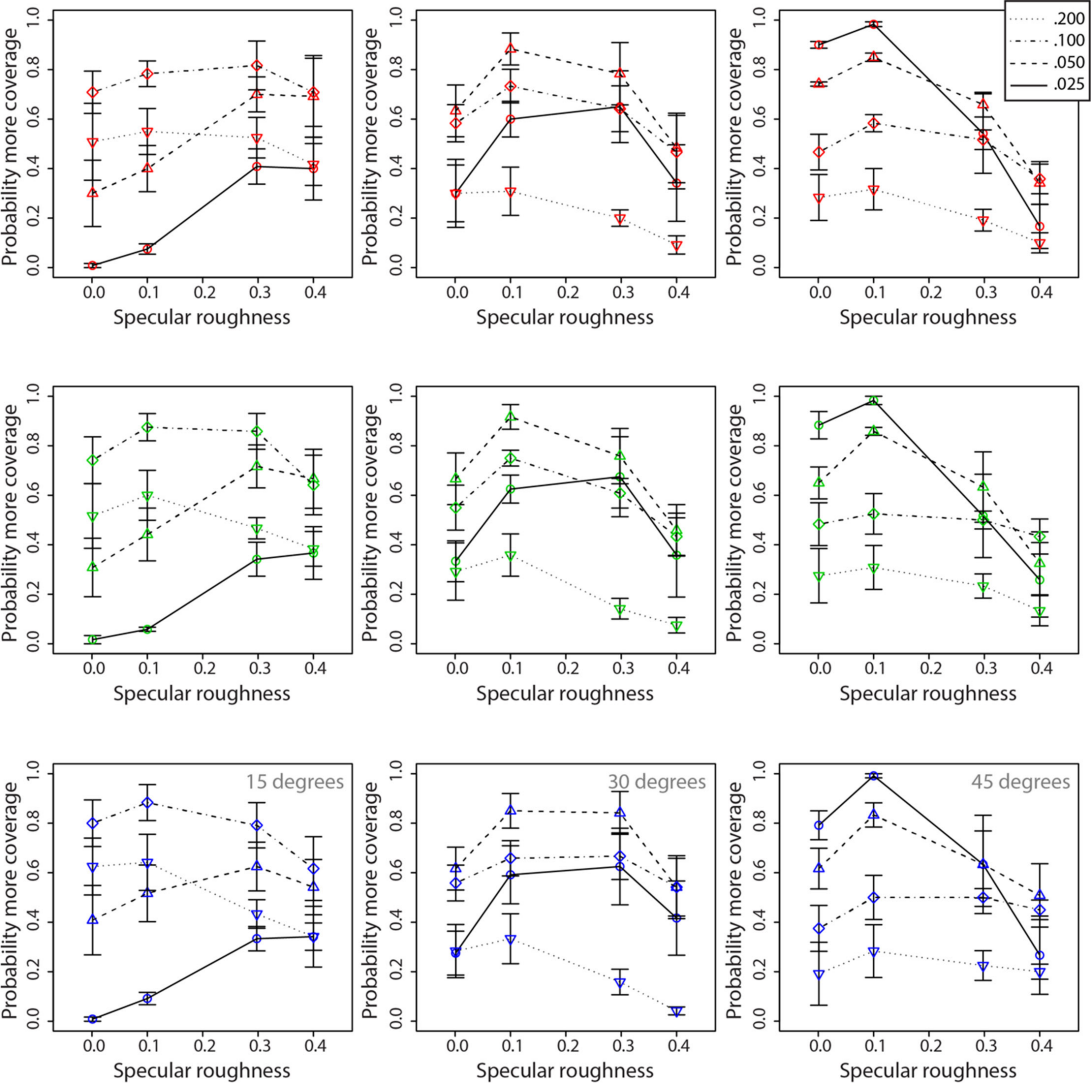
Means and standard errors for perceived coverage of surfaces by specular highlights across three different hues: red, green, and blue (top, middle, and bottom rows, respectively). Data plotted in each column represent the three surface orientations used in the experiment (15°, 30°, and 45°). Different line types and symbols are shown in the legend and correspond to data across the different relief heights.

We found the same consistent decline in perceived gloss with increasing specular roughness, especially for surfaces oriented more frontally to the observer and obliquely relative to the primary lighting direction, which other studies have reported (Marlow et al., 2012; Honson et al., 2020). We also found the same pattern of results as Honson et al. (2020) in that the effect of varying relief height on perceived gloss was more variable for surfaces that were oriented frontally (i.e., 15°). This can be observed in Figure 10, where there is larger displacement between curves plotted for the 15° condition compared to the increasingly slanted surfaces across all hues. An inverse pattern of responses across specular roughness values can also be observed for the lowest relief height value (0.025) across all hues (Figure 9); increases in perceived gloss were observed with increasing specular roughness values that asymptote between values 0.300 and 0.400. We found very little difference in the effects of relief height and specular roughness on perceived gloss across different hues. These results suggest that perceived gloss alone cannot account for the differences we observed in Experiment 1 for perceived lightness and saturation.

## Experiment 3

In Experiment 1, the magnitude of changes in perceived lightness and chroma were different across the three hues. Experiment 2 results suggest these differences are unlikely to be attributed to differences in perceived gloss, as percepts of gloss were somewhat invariant across changes in hue. For the 15° condition, there was a consistent decline in perceived gloss with increasing specular roughness in all relief heights except for the 0.025 condition, which had the opposite pattern—an increase in perceived gloss with increasing specular roughness that plateaued between 0.300 and 0.400. Given these findings, perceived gloss per se does not appear to account for differences in perceived color attributes (chroma and lightness) observed across the three different hues we considered.

An alternative explanation for perceived changes in lightness and chroma could be specular coverage (Honson et al., 2020). Increasing relief height from a flat plane will increase the local curvature of the surface, which in turn increases the range of surface normal orientations across the same surface regions. This increase in range of surface normal orientations will increase the number of surface regions with normals that bisect that angle between the viewing and illumination vectors, generating specular highlights. The variation in the distribution of specular highlights that results (specular highlight coverage) may potentially be used as a cue to differentiating diffuse from specular regions for assessing surface lightness and saturation. This specular coverage cue has been found in previous work to account for perceived gloss across a variety of viewing conditions (Marlow et al., 2012) and has been further extended to assess its covariance with perceived color saturation (Honson et al., 2020). In Experiment 3, we determined whether perceived specular coverage of our planar surfaces could account for the observed changes in perceived chroma and lightness with changes in specular roughness and relief height.

### Materials and methods

#### Observers

Four observers with normal or corrected-to-normal vision were recruited. All participants were authors of this study but were well trained in performing these psychophysical tasks. All procedures were approved for ethical clearance from Canon Information Systems Research Australia and followed the tenets of the Declaration of Helsinki.

#### Stimuli

All experimental stimuli were the same as those used in Experiment 2.

#### Procedure

The procedure for the current experiment was identical to the previous paired-comparisons experiment, except for a change in instruction. Here, the task posed to the observers was to “select which of the two images appears to have greater surface area covered by specular highlights.” Responses were recorded using the same procedures as used in Experiment 2.

#### Data analysis

Data were analyzed in the same way as described in Experiment 2. We computed probability estimates of perceived coverage for each image in each condition by dividing the number of times the image was selected as having more specular coverage by the number of times it was presented. We also performed Pearson's correlations to determine whether a weighted linear combination of perceived gloss and coverage estimates could account for perceived color attributes of lightness and chroma.

### Results and discussion

Perceived specular highlight coverage was plotted as a function of specular roughness for each of the levels of relief height and viewing orientations (Figure 10). Separate rows of axes show data obtained for surfaces having each of the three different diffuse colors. We analyzed the results separately for each of the nine conditions below.

Red surfaces were analyzed for effects on perceived highlight coverage at each viewing orientation using separate repeated-measures ANOVAs. For the 15° condition, there was no significant main effect of specular roughness, *F*(3, 9) = 1.28, *p* = 0.34, but there was a significant main effect of relief height, *F*(3, 9) = 17.05, *p* < 0.0005. There was also a significant interaction effect between specular roughness and relief height on perceived coverage, *F*(9, 27) = 4.54, *p* < 0.005. For the 30° condition, there was no significant main effect of specular roughness, *F*(3, 9) = 1.46, *p* = 0.29, but there was significant main effect of relief height, *F*(3, 9) = 24.78, *p* < 0.0005. There was no significant interaction effect between specular roughness and relief height on perceived coverage, *F*(9, 27) = 1.85, *p* = 0.11. For the 45° condition, there was a significant main effect of specular roughness, *F*(3, 9) = 19.02, *p* < 0.0005, and relief height, *F*(3, 9) = 14.38, *p* < 0.001. There was also a significant interaction effect between specular roughness and relief height on perceived coverage, *F*(9, 27) = 9.69, *p* < 0.00001.

Green surfaces were analyzed for effects on perceived highlight coverage at each viewing orientation using separate repeated-measures ANOVAs. For the 15° condition, there was no significant main effect of specular roughness, *F*(3, 9) = 0.86, *p* = 0.49, but there was a significant main effect of relief height, *F*(3, 9) = 43.87, *p* < 0.0001. There was also a significant interaction effect between specular roughness and relief height on perceived coverage, *F*(9, 27) = 9.69, *p* < 0.00001. For the 30° condition, there was no significant main effect of specular roughness, *F*(3, 9) = 2.64, *p* = 0.11, but there was a significant main effect of relief height, *F*(3, 9) = 17.23, *p* < 0.0005. There was a significant interaction effect between specular roughness and relief height on perceived coverage, *F*(9, 27) = 2.90, *p* < 0.05. For the 45° condition, there was a significant main effect of specular roughness, *F*(3, 9) = 5.43, *p* < 0.05, and relief height, *F*(3, 9) = 7.93, *p* < 0.01. There was also a significant interaction effect between specular roughness and relief height on perceived coverage, *F*(9, 27) = 6.48, *p* < 0.0001.

Blue surfaces were analyzed for effects on perceived highlight coverage at each viewing orientation using separate repeated-measures ANOVAs. For the 15° condition, there was no significant main effect of specular roughness, *F*(3, 9) = 0.23, *p* = 0.87, but there was a significant main effect of relief height, *F*(3, 9) = 43.76, *p* < 0.0001. There was also a significant interaction effect between specular roughness and relief height on perceived coverage, *F*(9, 27) = 9.46, *p* < 0.00001. For the 30° condition, there was no significant main effect of specular roughness, *F*(3, 9) = 1.33, *p* = 0.32, but there was a significant main effect of relief height, *F*(3, 9) = 17.23, *p* < 0.0005. There was a significant interaction effect between specular roughness and relief height on perceived coverage, *F*(9, 27) = 3.31, *p* < 0.01. For the 45° condition, there was no significant main effect of specular roughness, *F*(3, 9) = 2.18, *p* = 0.16, but there was a significant main effect of relief height, *F*(3, 9) = 8.71, *p* < 0.01. There was also a significant interaction effect between specular roughness and relief height on perceived coverage, *F*(9, 27) = 7.47, *p* < 0.0001.

Across all three hues, we found the same complex, systematic differences in perceived specular coverage across different orientations, specular roughnesses, and relief heights as Honson et al. (2020). With low relief height values, coverage was predicted to be incrementally greater with increasing the orientation of the stimulus away from the observer and toward the light source. With high relief height, the opposite was predicted, whereby coverage would be incrementally lower with increasing surface orientation away from the observer and towards the light source. Although these predictions are supported by the pattern of responses we observed in the present study, the magnitude of curves for relief heights across specular roughness values seem to differ quite considerably between the present experiment and Honson et al. (2020). For example, the curve plotting values for a relief height of 0.200 at an orientation of 15° had the highest probability of being chosen to have higher coverage in Honson et al. (2020) (about 75% of the time) but was chosen only about 50% of the time in the present experiment. These differences in magnitude may be reflective of the lightness and chroma values chosen for the stimuli in the present experiment (60 and 60, respectively), which were considerably lower compared to the values used in Honson et al. (2020) (71.679 and 101.237, respectively).

We found that relief height and specular roughness generated consistent variations in perceived specular highlight coverage across hues. Similar to Honson et al. (2020), we observed an interaction effect demonstrated by the apparent traveling wave in the plots across surface orientation conditions. The amount of perceived specular coverage tended to increase with increasing specular roughness for the 15° viewing condition but decreased with increasing specular roughness for the 45° viewing condition. Increasing relief height increased coverage for the 15° condition where coverage was initially very low but reduced it for the 45° orientation, where initial coverage was higher. The strong interaction effects we found here were consistent across the different hue conditions.

Despite the similarity across hues in the effects of surface properties on perceived gloss (Experiment 2) and specular highlight coverage (Experiment 3), we attempted to ascertain whether some weighted linear combination of perceived gloss and coverage could account for the variations in perceived chroma and lightness we observed in Experiment 1. This analysis was based on the three observers who participated in these three relevant experiments, all of whom were authors (ZJI, QH-T, MA). We separately fit the following weighted linear models:

$$\begin{array}{c} P_{\mathrm{saturation}}^{'}=W*P_{coverage}+\left(1-W\right)*\left(1-P_{gloss}\right)\\ P_{\mathrm{lightness}}^{'}=P*P_{coverage}+\left(1-W\right)*\left(1-P_{gloss}\right) \end{array}$$

where *P*′*_saturation_* and *P*′*_lightness_* refer to the estimated perceived saturation and lightness, respectively, for *P_coverage_* and *P_gloss_*, corresponding to the perceived coverage and gloss estimated through psychophysical experiments (Experiments 2 and 3). The model weight (*W*) was estimated empirically based on linear least-squares fits between the model output and actual psychophysical data of perceived saturation and lightness. We allowed the weight of the model to vary parametrically between –1 and 1 at 0.01 steps to identify the correlation that accounted for the most variability in perceptual data on saturation and lightness. A value for *W* of 0 indicates no weighting on perceived coverage and 100% on perceived gloss. A value for *W* of 1 indicates 100% weighting on perceived coverage and no weighting on perceived gloss. For example, if surfaces are perceived as glossier, then it is assumed that the visual system has intrinsically attributed more of the specular image structure to glossiness. If the visual system attempts to attribute image content to physical causes in this way (Barrow & Tenenbaum, 1978), then the perception of color saturation and lightness should be less biased by residual specular image structure not attributed to glossiness.

**Figure 11.**
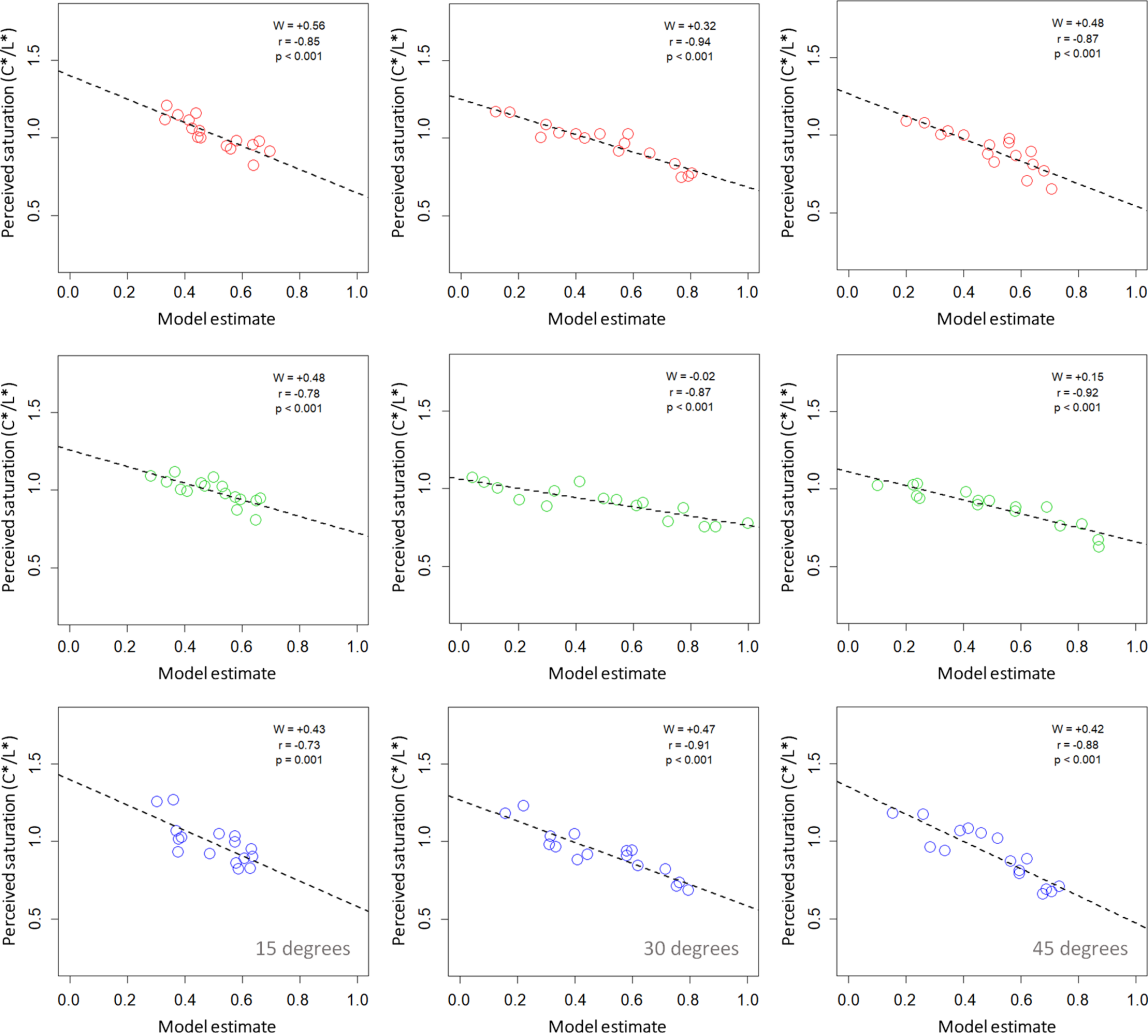
Mean perceived saturation (C*/L*) plotted as a function of our weighted linear model based on perceived coverage and gloss. Results are shown for the three different hues: red, green, and blue (top, middle, and bottom rows, respectively). Data plotted in each column represent the three surface orientations (15°, 30°, and 45°). Legends show the weighting (*W*) for the best model fit with the associated correlation coefficient and *p* value. Data are based on averages across three observers for 16 conditions pooled for each plot (four levels of relief height × four levels of specular roughness).

Figure 11 shows the correlations for all surface colors and viewing orientations between perceived saturation (based on C*/L* results of Experiment 1) and a weighted linear combination of perceived gloss (Experiment 2) and specular highlight coverage (Experiment 3). Data were averaged across all participants within each of the three experiments. Data for different relief heights and specular roughness levels were then pooled to assess the performance of model predictors in each of the nine plots. The legend in each plot shows the value of *W* (with the correlation coefficient and *p* value) that was found to account for the greatest variability in perceived saturation. The average model across all conditions pooled returned a weight of *W* = 0.41, weighting gloss 59% and coverage 41%. Eyeballing the separate conditional plots suggests that model weights for the blue hue were similar to the average model response. Variability in model weighting was greater for the red surfaces and greatest for green surfaces. All viewing orientations and surface colors generated a high level of prediction of perceived saturation using the optimal weighted linear combination of perceived gloss and coverage. Indeed, in Experiment 1, we found consistent dependence of perceived saturation on relief height and specular roughness for these conditions.

On close inspection of the data pooled across all conditions, the correlation between perceived coverage and gloss was found to be relatively low, though significant (*r* = 0.32, *p* < 0.0001). Using these pooled data, we found that the optimum weight for fitting our linear model to account for perceived saturation was *W* = 0.41 (*r* = –0.77, *p* < .000001), which is very close to the weights required to optimally fit responses to estimates of perceived saturation of blue surfaces across each viewing orientation. Forcing the weight to 0.41 in all subconditions resulted in slightly different fits, as shown in Table 2. We found that the blue surfaces generated the same model correlations when optimized using a variable *W* or when forced to the 0.41 weighting. Red and green surfaces generated relatively high correlations across all conditions, but fits were consistently lower when forced to the 0.41 weighting. All correlations were significant (*p* ≤ 0.001). The results of simple linear regression models suggest that both gloss and coverage together are useful for explaining the perception of color saturation across hues, with emphasis on gloss over coverage.

**Table 2.** Model fits for perceived saturation using weights for individual and pooled data.

Hue	Slant	*W*	*r_W_*	*r*_0.41_
Red	15°	+0.56	–0.85	–0.75
	30°	+0.32	–0.94	–0.93
	45°	+0.48	–0.87	–0.85
Green	15°	+0.48	–0.78^*^	–0.75^*^
	30°	−0.02	–0.87^*^	–0.77^*^
	45°	+0.15	–0.92^*^	–0.84^*^
Blue	15°	+0.43	–0.73^*^	–0.73^*^
	30°	+0.47	–0.91^*^	–0.91^*^
	45°	+0.42	–0.88^*^	–0.88^*^

* Significant correlation (*p* ≤ 0.001).

**Figure 12.**
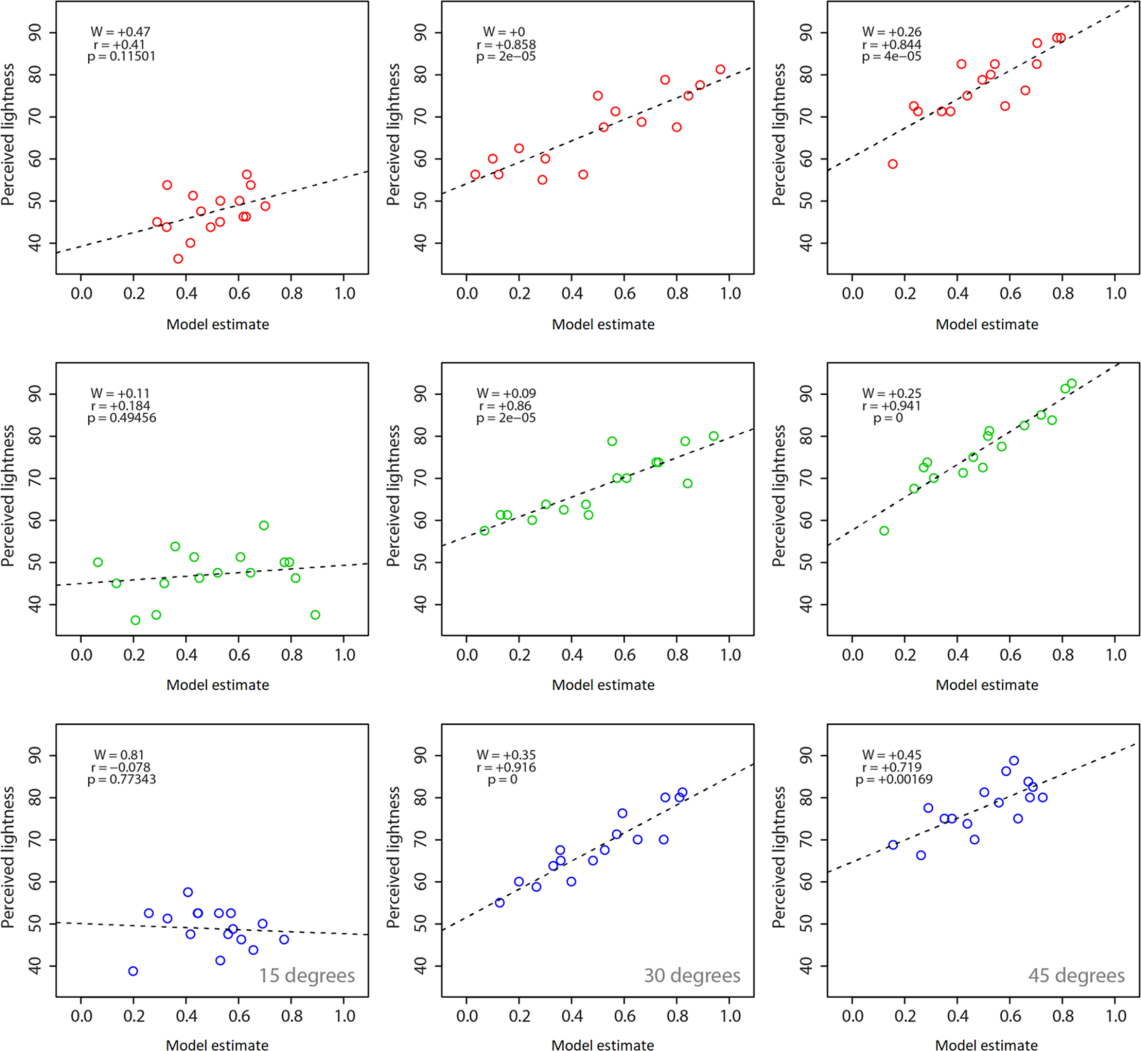
Mean perceived lightness plotted as a function of our weighted linear model based on perceived coverage and gloss. Results are shown for the three different hues: red, green, and blue (top, middle, and bottom rows, respectively). Data plotted in each column represent the three surface orientations (15°, 30°, and 45°). Legends show the weighting (*W*) for the best model fit with the associated correlation coefficient and *p* value. Data are based on averages across three observers for 16 conditions pooled for each plot (four levels of relief height × four levels of specular roughness).

We also considered the ability of our model to account for perceived lightness. Figure 12 shows the correlations for all surface colors and viewing orientations between perceived lightness (based on results of Experiment 1) and a weighted linear combination of perceived gloss (Experiment 2) and specular highlight coverage (Experiment 3). Here, the model was found to account very well for the perception of lightness across all the three different surface colors we used. Significant correlations were found for conditions where surfaces were oriented at 30° or 45° only. No significant correlations were found between perceived lightness and our weighted linear model for the 15° viewing condition. When we forced values to the optimum model fit to the pooled data (*W* = 0.34), there was little change in the overall strength of the model correlations with perceived lightness across hues (see Table 3).

**Table 3.** Model fits for perceived lightness using weights for individual and pooled data.

Hue	Slant	*W*	*r_W_*	*r*_0.34_
Red	15°	+0.47	+0.41	+0.38
	30°	0	+0.86^*^	+0.78^*^
	45°	+0.26	+0.84^*^	+0.84^*^
Green	15°	+0.11	+0.18	+0.17^*^
	30°	+0.09	+0.86^*^	+0.82^*^
	45°	+0.25	+0.94^*^	+0.93^*^
Blue	15°	+0.81	–0.08	+0.02^*^
	30°	+0.35	+0.92^*^	+0.92^*^
	45°	+0.45	+0.72^*^	+0.70^*^

* Significant correlation (*p* ≤ 0.005).

The model generated significant negative correlations with the perceived saturation of all three hues for surfaces viewed at 15° (*p* ≤ 0.001). Although the model accounted for perceived lightness at more oblique viewing orientations, this was not the case in the more frontal viewing condition of 15°. Model predictions were therefore more reliable in predicting saturation than lightness. Hence, despite the variability in perceived color saturation found in Experiment 1, we found here that a weighted linear combination of gloss and specular coverage largely accounts for the appearance of color saturation. Emphasis on gloss and coverage was more consistent for blue hues, as greater dependence on gloss was found in green conditions. We examine the broader implications of these findings further below.

## General discussion

The present study sought to investigate and model how hue, surface orientation, and mesoscopic and microscale shape can affect the perceived lightness and saturation (estimated from chroma and lightness) of an object. To this end, we generated planar surfaces and varied the surface hue, the orientation of the surface toward the light source (from above), and the mesoscopic relief height and specular roughness. Under each of these conditions, we parametrically varied specular roughness.

In Experiment 1, we found that perception of the lightness and saturation of an object could be biased by changes imposed in the orientation of object to the light source and its surface properties. The magnitude at which the perceived lightness of a surface increased and saturation decreased systematically varied as a function of the parameters we manipulated. Perceived saturation declined, and perceived lightness increased with increasing specular roughness, replicating the findings of previous studies using HSV color matching (Honson et al., 2020). Differences in the magnitude of these effects were observed across the three different hues we tested (red, green, and blue), whereby the magnitude in which perceived saturation decreased across specular roughness was greatest for the shorter wavelength blue hue. These effects do not appear to be attributed to ordinal differences in luminance, as we roughly equated luminance across the three hues, and the mean luminance of blue surfaces was close to the luminance of red and green surfaces. However, there were strong dependencies of perceived lightness on specular roughness across the three hue conditions. The magnitude of the difference in perceived lightness and saturation observed for blue, but not red and green, may indeed be due to perceptual biases that lie along the blue/yellow axis (Churma, 1994; Pearce et al., 2014; Winkler et al., 2015; Weiss et al., 2017).

Given that blue and yellow are the dominant illuminant colors along the daylight locus, it is thought that the visual system has specific priors that account for or discount the color of illuminants along this locus when making color judgments. It has indeed been found that color constancy is worse under blue illumination (Pearce et al., 2014; Winkler et al., 2015) and that bluish tints are attributed to the illuminant rather than the object itself (Churma, 1994; Pearce et al., 2014; Winkler et al., 2015). Along these lines of reasoning, we propose that the specular highlights in our blue tile stimuli are more difficult to segment and therefore may be attributed to the illuminant rather than the color of the tile per se. Due to this inability to disentangle specular highlights from tile hue, lightness judgments can be artificially inflated (especially for tiles with low relief heights), as lightness judgments have been found to be based on the brightest part of the object itself (Toscani, Valsecchi, & Gegenfurtner, 2013).

Interestingly, despite the fact that specular coverage estimates across all three hues were essentially the same (Experiment 3), the fact that saturation and lightness judgments for blue significantly differed from red and green suggests that these judgments are not influenced by top-down knowledge. Rather, the bias for blue we observed is bottom up and cannot be overwritten—at least in the case of our experimental manipulation(s). It would be interesting in future work to see if we would observe similar sorts of biases for yellow tiles, as the visual system has evolved not only to blue illuminants along the daylight locus but also to yellow illuminants. It is possible that we would observe a different pattern of results, as it has been demonstrated that yellow illuminants are more strongly attributed to the hue of objects in a scene compared to blue illuminants (Winkler et al., 2015). However, more research is necessary to extend these findings to more complex stimuli such as the stimuli used in the present study.

Although we did not test many different hues to assess whether any effect of changing hue is continuous or categorical, research on constancy suggests that hue effects may involve a gradual continuous profile (Weiss et al., 2017). That study found that color constancy was preserved at the borders of different color categories, rather than generating large differences relative to the center of categorical zones. It would, however, be useful to explore the potential effects of color we report across a wider range of hues.

In Experiment 2, we found that perceived gloss generally declined with increasing specular roughness across all viewing orientations and relief heights. Similar to Honson et al. (2020), the only condition that followed the inverse was the low relief height viewed at the 15° viewing angle. There were no clear differences in the pattern of perceived gloss data across the three different surface hues, suggesting that additional information would be required to account for the differences in perceived lightness and saturation we observed across hues in Experiment 1. Hence, Experiment 3 considered the role of perceived specular highlight coverage. We obtained estimates of perceived specular highlight coverage from the same observers who participated in Experiment 2. We observed a pattern of data that can be described as a traveling wave with increasing surface orientation away from the viewing direction. The wave consisted of a displacement in peak perceived coverage toward lower specular roughness at more oblique viewing orientations. This wave-like pattern was primarily observed for low relief heights. Greater relief heights tended to maintain an inverted U-shaped pattern of perceived coverage across changes in surface orientation.

Applying a weighted linear regression model similar to that used in Honson et al. (2020) to our data, we found that perceived coverage and gloss could account very well for the perceived lightness of surfaces viewed at oblique angles of 30° and 45°. However, the weighted linear model performed better at accounting for perceived saturation at more frontal viewing orientation of 15°. These complex dependencies of perceived saturation and lightness on viewing orientation were on the whole observed across the different hues used to render the diffuse component of our 3D planar surfaces. Hence, we found that perceived lightness and saturation depend not only on viewing orientation but also on surface properties of hue, relief height, and specular roughness.

Although perceived gloss and coverage measurements are essentially very similar across hues, these properties may be weighted differently across hues when judging the perceived saturation and lightness of our surface stimuli. For example, the model weighting on gloss and coverage was found to be generally diagnostic of perceived saturation across hues but was found to emphasize gloss more for green surfaces. The model weighting was more consistent for blue surfaces. This result suggests that additional information may be useful for constraining perceived saturation for green surfaces, which may not be the case for blue surfaces. This finding may suggest that different priors may be at play when judging saturation and lightness (e.g., difficulty parsing surface hue from the illuminant) compared to gloss and surface coverage judgments. Irrespective of the differences, however, the variations in perceived lightness and saturation appear to be largely explained by perceptual indices of perceived gloss and specular highlight coverage.

The apparent variability in the model explanatory power of gloss and specular coverage over perceived lightness across different hues might be attributed to the separability of specular and diffuse shading components (Anderson & Kim, 2009). Indeed, surfaces with higher specular roughness were estimated to have higher lightness and lower saturation than surfaces with sharp specular reflections. The blurring of specular edge contours appears to have made it perceptually challenging to separate the diffuse component from the specular component for color saturation and lightness analysis. Indeed, we found that the increasing specular roughness generally increased perceived lightness and reduced perceived saturation. In support of the view that the visual system separates diffuse from specular shading for color perception, evidence suggests that surface regions covered by specular highlights are ignored when participants estimate the body color or lightness of a surface (Kim, Marlow, & Anderson, 2012; Toscani, Valsecchi, & Gegenfurtner, 2017). The co-dependence of perceived saturation on not just perceived gloss but also perceived specular coverage supports the view that participants selectively attend to surface regions that are not covered in specular highlights when estimating color saturation and lightness. Due to the tendency of specular highlights to appear near brighter regions of diffuse shading in natural scenes (Kim, Marlow, & Anderson, 2011), observers will tend to be biased toward estimating color using darker regions of diffuse shading (Toscani et al., 2013; Toscani & Valsecchi, 2019). Consistent with this view, we found that surfaces with greater relief height were perceived as darker overall compared with surfaces with lower relief height, presumably due to the greater variability in orientation of surface normal pointing away from the primary lighting direction.

In earlier work, Marlow et al. (2012) found that perceived gloss could be explained by a weighted linear combination of specular sharpness, contrast, and coverage in monocular images. The model we used in the present study was proposed to explain the perceived lightness and saturation of surfaces, which was assumed to depend on the (in-)ability of the brain to separate diffuse from specular content. Our model was successful in accounting for variations in perceived lightness (and saturation). It is possible that the model could be improved by obtaining additional data on perceived specular sharpness and contrast (in addition to coverage). However, this approach involves a purely perceptual model that requires psychophysical information to be obtained from human observers on the appearance of the specular properties of surfaces they see in images. It would be useful to improve the utility of this model by automating the measurement of specular highlight coverage and other specular attributes computationally.

In summary, we found complex dependencies of perceived saturation and lightness on the physical properties of surfaces. Although some of the differences might be attributed to residual inaccuracies in representing LCH positions in sRGB space, our findings show there exists complex nonlinear perceptual dependencies of perceived lightness and saturation on (1) the intended hues of 3D surfaces rendered, (2) surface orientation relative to the light source, (3) mesoscopic surface relief height, and (4) microscale specular roughness. It would be beneficial in future research to determine what aspects of 3D rendering and the simulated lighting environment might best account for these perceptual variations so that the representation of color (and the perception of it) can be most appropriately managed.

## Supplementary Material

Supplement 1

Supplement 2
