# Supplementary figures and images for: Surface properties and the perception of color
Source: J Vis. 2021 Feb 12;21(2):7. doi: 10.1167/jov.21.2.7 (PMC7888285; doi:10.1167/jov.21.2.7)

## Appendix A

Red 15°

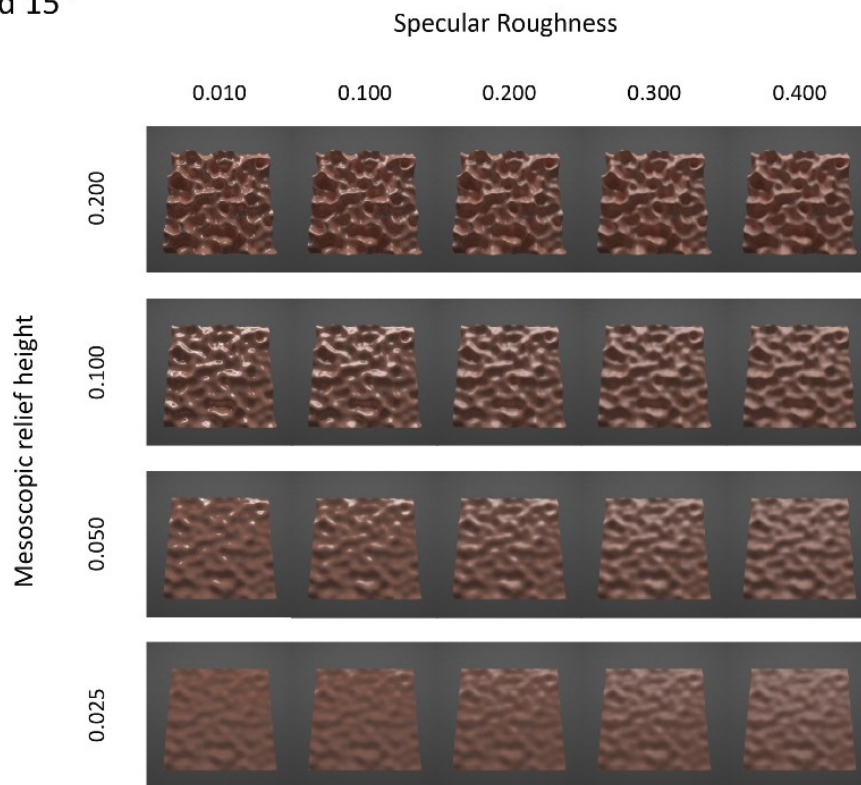

Red 30°

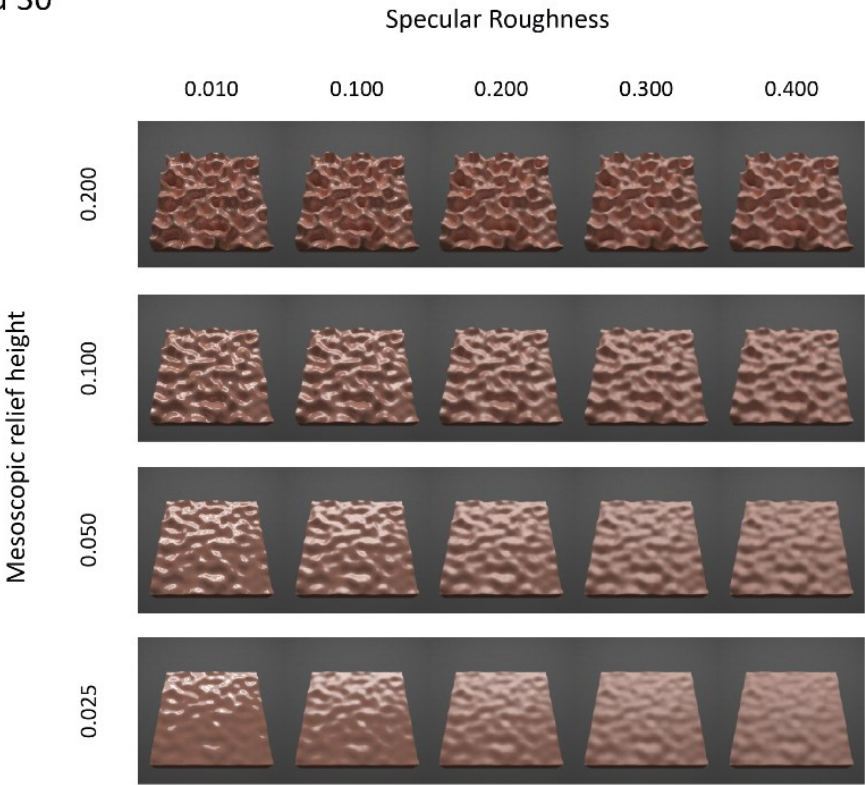

Red 45°

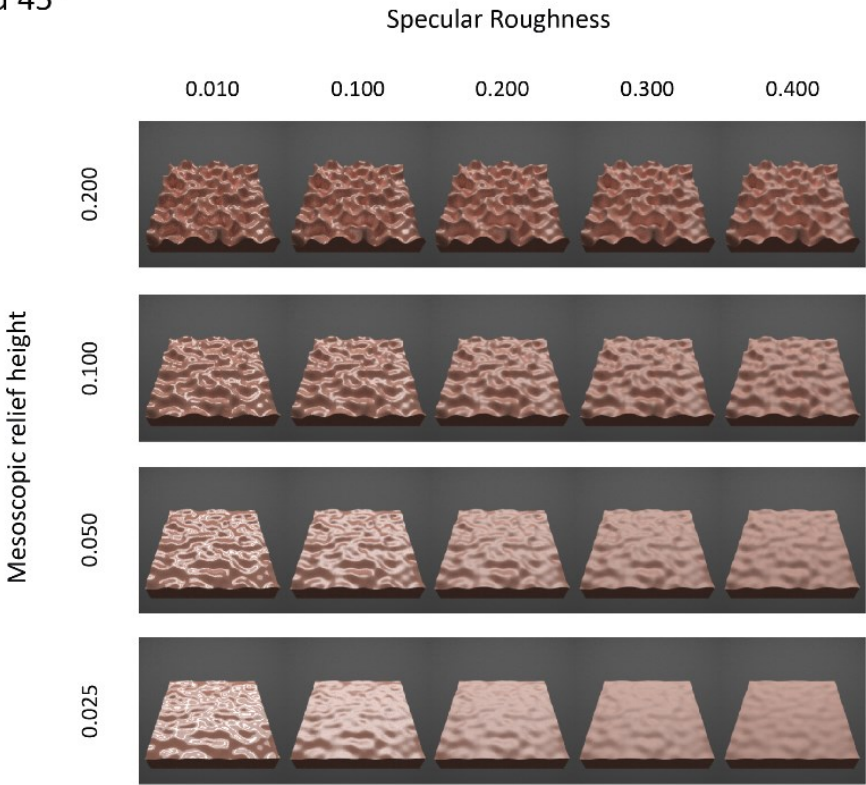

Green 15°

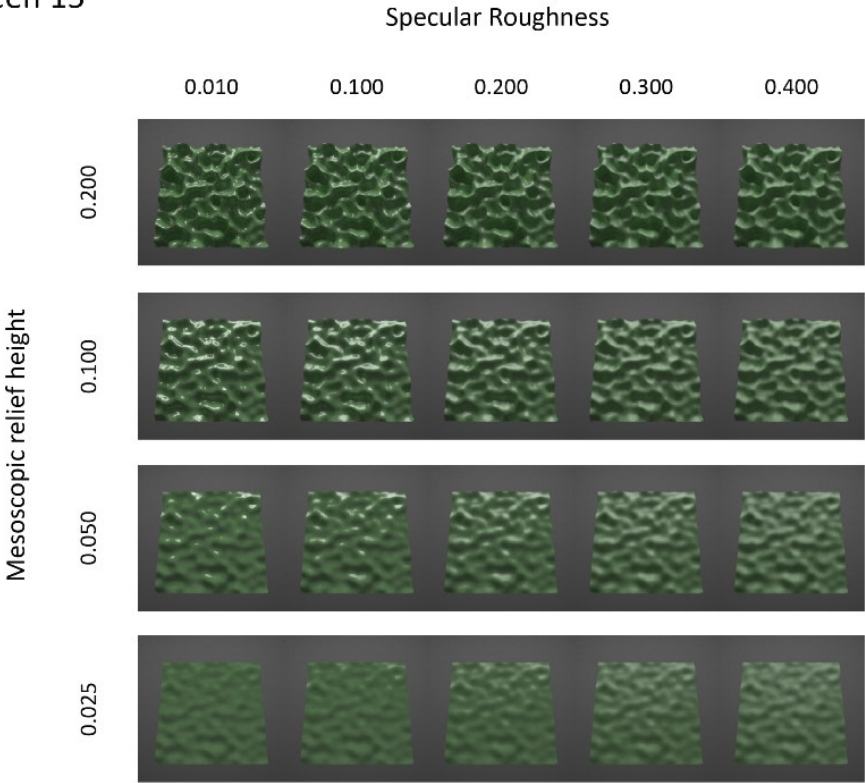

Green 30°

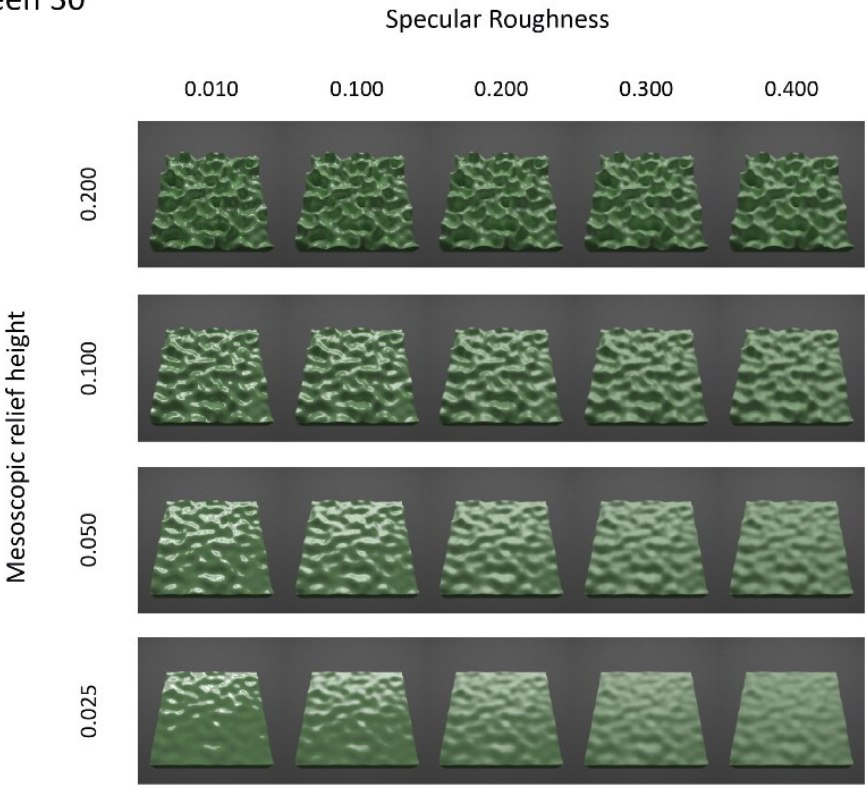

Green 45°

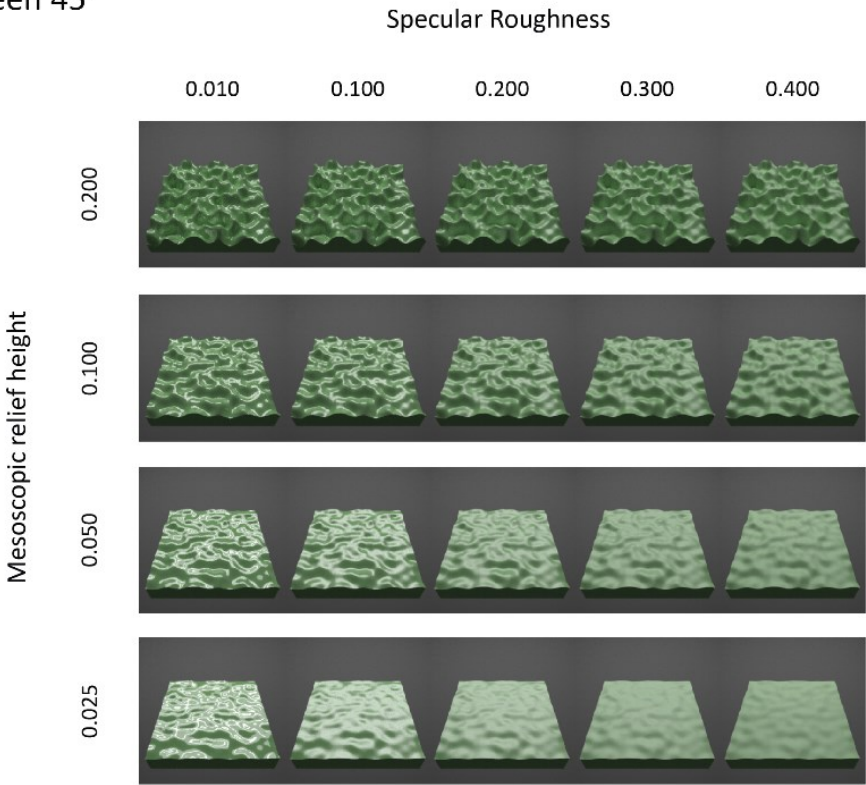

Blue 15°

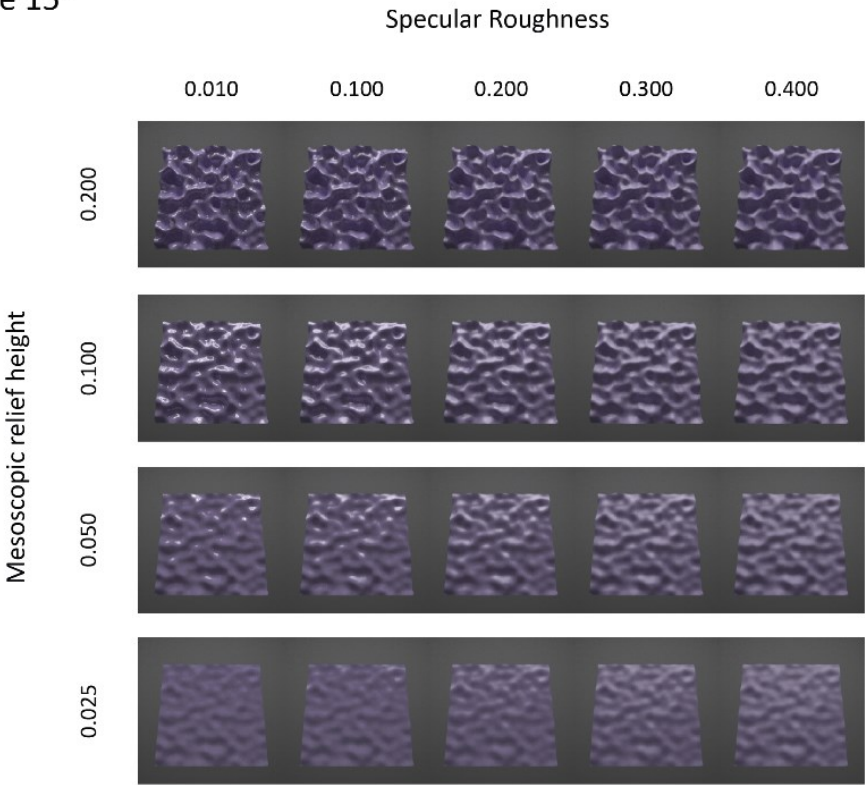

Blue 30°

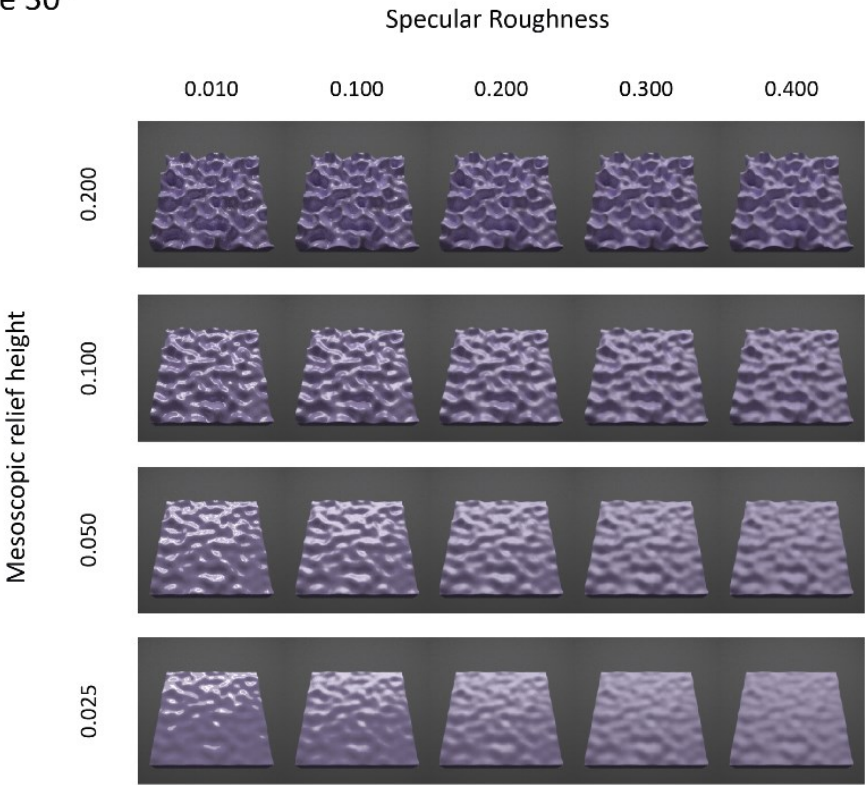

Blue 45°

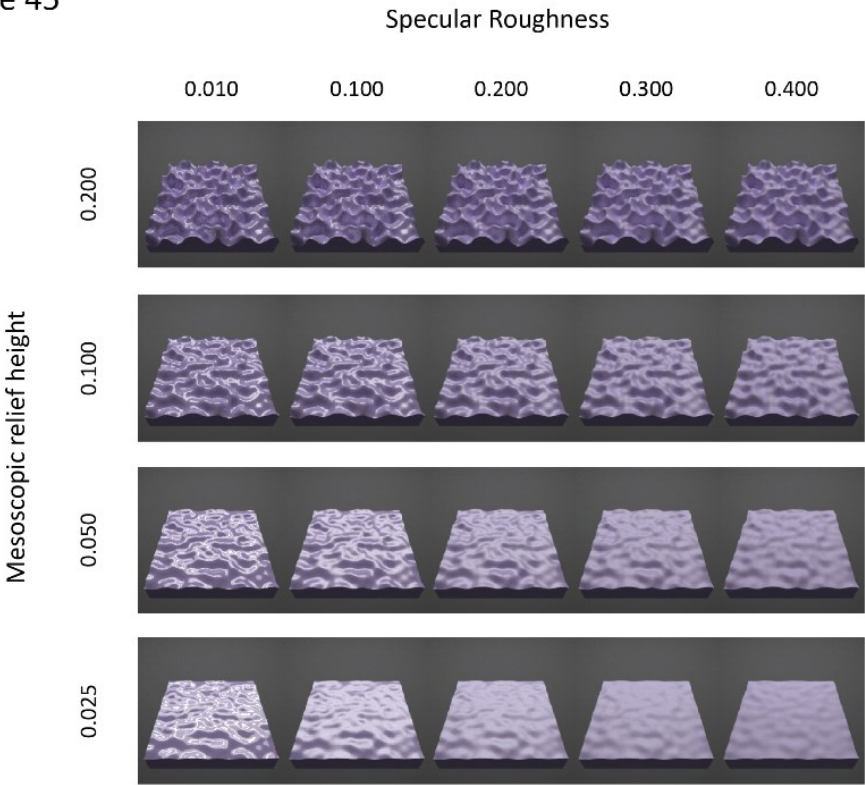

Supplement: Supplement 1 [file jovi-21-2-7_s001.pdf]
