# Supplementary material for: Surface properties and the perception of color
Source: J Vis. 2021 Feb 12;21(2):7. doi: 10.1167/jov.21.2.7 (PMC7888285; doi:10.1167/jov.21.2.7)
Supplement: Supplement 2 [file jovi-21-2-7_s002.pdf]

Appendix B

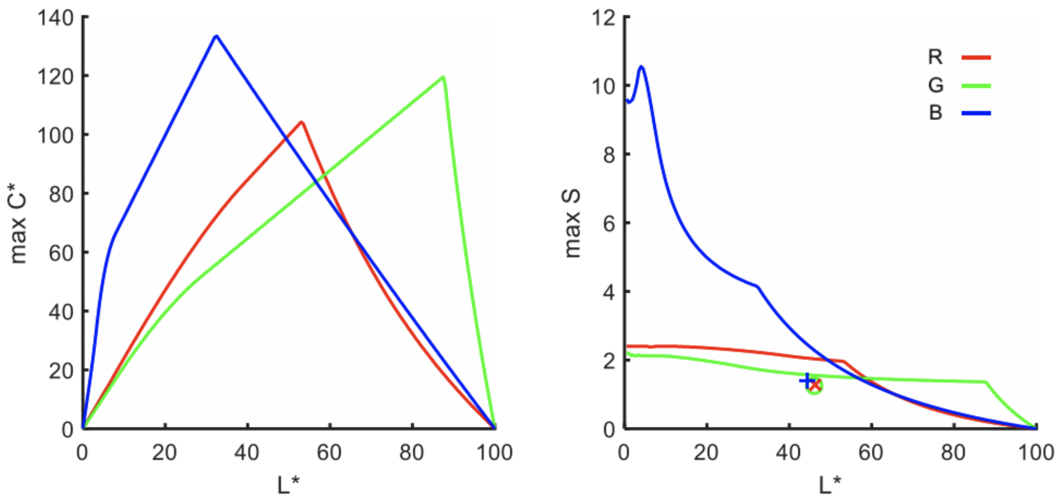

Supplementary Figure B1. Plots of sRGB gamut limits for a wide range of L* and C* values across the three hues used in the present study. Data points in each plot represent RGB triplet values between 0 and 1 after LCH conversion. Left panel: C* limit (max C*) for each L* level for the three hues. Right panel: saturation limit (max S) as a function of L* for the three hues. The maximum saturation settings derived from the data in Figure 8 are also plotted with corresponding L* data derived from Figure 9. The 'o' represents the green hue, the 'x' represents red, and the '+' represents blue.
